# First thorough assessment of de novo oocyte recruitment in a teleost serial spawner, the Northeast Atlantic mackerel (*Scomber scombrus*) case

**DOI:** 10.1038/s41598-021-01234-1

**Published:** 2021-11-08

**Authors:** Thassya C. dos Santos Schmidt, Anders Thorsen, Aril Slotte, Leif Nøttestad, Olav S. Kjesbu

**Affiliations:** Institute of Marine Research, PO Box 1870 Nordnes, 5817 Bergen, Norway

**Keywords:** Marine biology, Population dynamics

## Abstract

The understanding of teleost fecundity type (determinate or indeterminate) is essential when deciding which egg production method should be applied to ultimately estimate spawning stock biomass. The fecundity type is, however, unknown or controversial for several commercial stocks, including the Northeast Atlantic mackerel (*Scomber scombrus*). Aiming at solving this problem, we applied state-of-the-art laboratory methods to document the mackerel fecundity type, including any de novo oocyte recruitment during spawning. Initially, active mackerel spawning females were precisely classified according to their spawning status. The number and size of all phase_i_-specific oocytes (12 phases), with a special attention to previtellogenic oocytes phases (PVO [PVO2 to PVO4a–c]), were also thoroughly investigated. Examinations of relative fecundity (RF_i_) clarified that the latest phase of PVOs (PVO4c) are de novo recruited to the cortical alveoli–vitellogenic pool during the spawning period, resulting in a dome-shaped seasonal pattern in RF_i_. Hence, we unequivocally classify mackerel as a true indeterminate spawner. As PVO4c oocytes were currently identified around 230 µm, mackerel fecundity counts should rather use this diameter as the lower threshold instead of historically 185 µm. Any use of a too low threshold value in this context will inevitably lead to an overestimation of RF_i_ and thereby underestimated spawning stock biomass.

## Introduction

The population egg abundance divided by individual fecundity is used in many regions of the world’s ocean to provide indices of spawning stock biomass (SSB) of commercially important teleosts—so-called Egg Production Methods (EPMs)^1,2^. However, one of the main challenges is to determine the kind of fecundity type in question and what it implies in terms of oocyte production during the spawning period, often found to be an evasive reproductive trait spanning theoretically from truly “fixed” (determinate) to truly “unfixed” (indeterminate)^3–5^. In determinate species the fecundity can be given prior to spawning, whereas in indeterminate species this is clearly not the case since new (de novo) oocytes are recruited to the maturing (cortical alveoli–vitellogenic) pool to be subsequently spawned as eggs^1,3,6^. Based on this dichotomic fecundity type, separate methods have been used within EPMs^7^. The annual egg production method (AEPM)—normally used on species with determinate fecundity—considers the total number of vitellogenic oocytes prior to the onset of spawning after correction for atretic loss^7^. The main alternative method, the daily egg production method (DEPM)—principally designed for species with indeterminate fecundity—takes into consideration the batch fecundity and spawning fraction (and sex ratio as in AEPM), normally at the peak of spawning^3,7^. One of the established criteria used to distinguish the fecundity type is the presence of a hiatus (or gap) in the oocyte size frequency distribution (OSFD)^4,6^. Formally, a clear hiatus should be found between previtellogenic (PVO) and vitellogenic oocytes (VO) in determinate spawners whereas being absent in indeterminate spawners^5,8,9^. This distinction may, however, be met with interpretation problems, e.g. the assumed determinate Atlantic cod (*Gadus morhua*) shows individual examples of OSFD with missing or indistinct hiatus^10^, in the indeterminate horse mackerel (*Trachurus trachurus*) the hiatus first emerges in late spawners^11^, whilst hiatus formation (cortical oocyte growth) in the indeterminate round herring (*Etrumeus teres*) coincides with hydration of the advanced oocyte cohort^12^. Neverthless, for many years, “the hiatus method”^10^ has been used as a semiquantitative tool to decide upon the fecundity type, though most research consortia focus in these respects on the VOs rather than the PVOs, the latter an emerging field^13,14^.

So, to move forward on this challenging topic, i.e. in terms of accurate and precise assessment of de novo oocyte recruitment, two “sharp tools” need to be in place, both with reference to the microscopic (cellular) level: 1) a reliable method for quantification of the various, multiple phases (“waves”) of oocytes involved, and 2) a true classification method of where the given female resides in the spawning period—whether it being early, mid or late to properly handle the time aspect, i.e., to qualify as a production estimate. Thus, any associated macroscopic staging classification (see below) was in this particular situation considered too imprecise and thereby debateable^13^. This article aims at resolving this de novo oocyte recruitment assessment problem by merging state-of-the-art techniques, represented by the oocyte packing density (OPD) theory^15^—providing phase-specific oocyte numbers—and the most recent ultrametric method^10^—detailing the “stage of spawning”^4,16^ represented by the oocyte ratio category. In short, earlier PVO and resulting de novo recruitment investigations have lacked these detailed insights, which are paramount to reconstruct the oocyte dynamics in the target species. If succeeding in these regards, today’s strict definitions of dichotomic fecundity types should become of less importance, e.g. one might adjust for advanced PVO production and the following de novo recruitment to the vitellogenic pool when using the AEPM on an indeterminate spawner.

Northeast Atlantic mackerel (*Scomber scombrus*, hereafter referred to as mackerel), our study model, has a controversial fecundity type—rooted in the unclear de novo recruitment^4^ speaking for the optional application of either the AEPM or the DEPM^7^. The mackerel fecundity type was comprehensively investigated by Greer-Walker et al.^4^, using a total of eight criteria—partly adopted from Hunter et al.^17^—among them any presence of a hiatus between PVOs and VOs, variation in oocyte number and diameter during the course of spawning, VO growth rate, and trend in atresia presence. According to these authors, the mackerel fecundity should be classified as determinate “for all practical purposes”, even though some ovarian development features evaluated were typical of an indeterminate spawner species^4^. In view of this statement and related elaborations, the fecundity of mackerel has been triennially estimated since 1977 by the AEPM^18^, although an attempt to implement the DEPM method was undertaken in the 1989 season^19^. Since 2013, the DEPM has been tested during this ICES Triennial Mackerel and Horse Mackerel Egg Survey and the first results just presented^18^, detecting, among other aspects, a major interannual variability in batch fecundity (number of eggs/batch) and spawning fraction^18^.

Based on the outlined controversy about the mackerel reproductive strategy and important discrepancies in estimates among primary stock assessment methods^20^, we combined OPD theory, applied previously on both determinate^15,21^ and indeterminate species^15,22–25^ with the new ultrametric method^10^ to clarify whether the mackerel fecundity type is determinate or indeterminate, or an in-between combination of both fecundity types^4^. Ultimately, we accurately quantified how many eggs each mackerel de facto releases over the season by adding in the advanced, relevant PVO production. The methodological routes taken along with the refreshed conceptual framework regarding fecundity type should be transferable to oviparous teleosts in general, and, possibly, other marine ectotherms exhibiting related reproductive strategies.

## Results

### Oocyte size frequency distribution

The OSFD, based on wholemount analysis (formalin-preserved diameter measurements), did not show any hiatus between the assumingly largest PVOs and the smallest VO (Supplementary, Fig. S1). The corresponding mean threshold value, determined statistically by the Gamma/Gaussian method (see technical details below), was 192 µm (95% CI: 187–196 µm) (Supplementary, Fig. S1). Based on histology, this value was, however, at ~ 230 µm, i.e. the formalin-preserved oocyte diameter of PVO4c (Supplementary, Figs. S2B, S3, Table S1).

### Spawning progress

**Figure 1:**
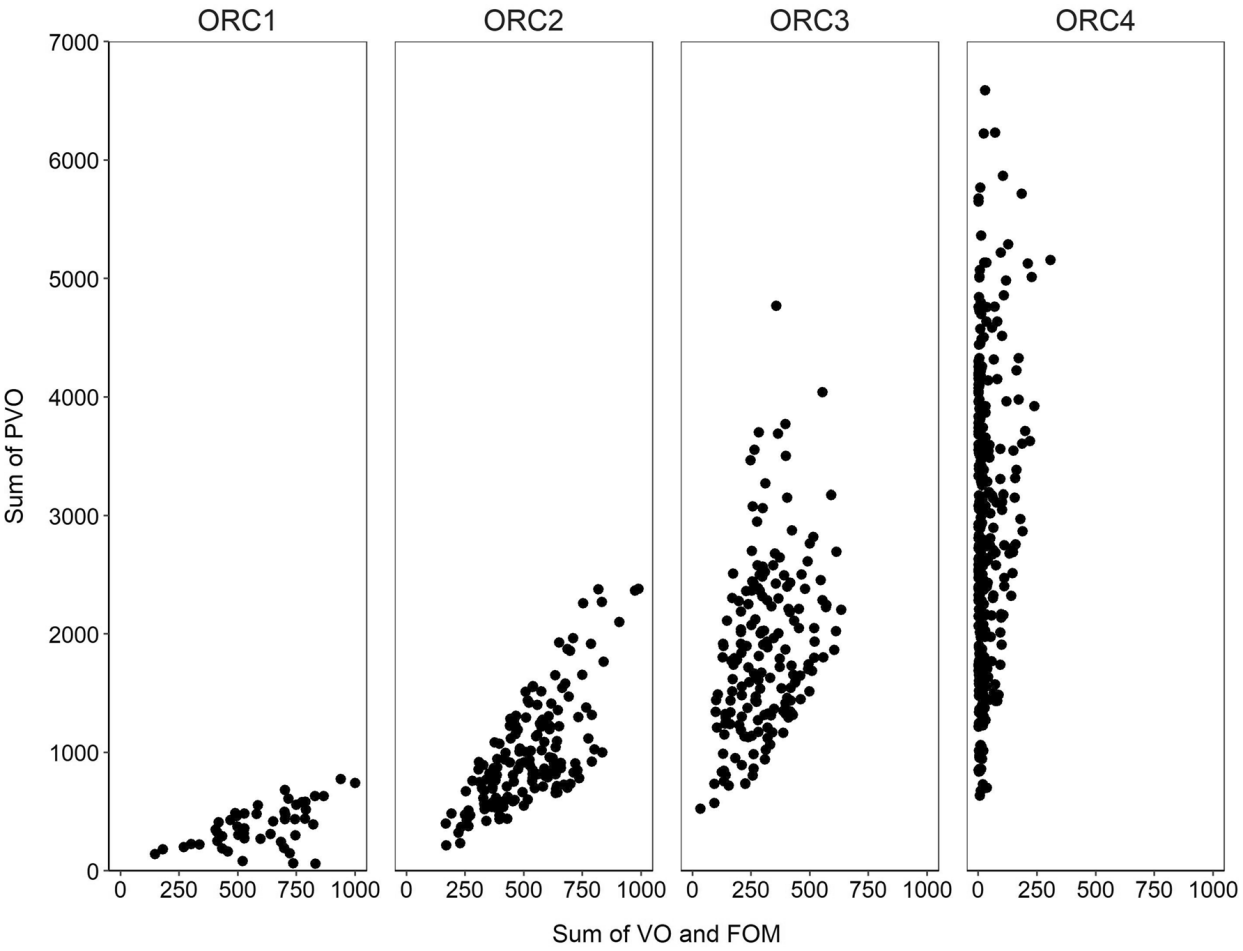
Wholemount counts of previtellogenic (PVO) versus developing oocytes (VO and FOM) used within the ultrametric method to categorize the “stage of spawning” represented by the oocyte ratio category (ORC). The resulting ORC category (ORC1-4) is showed above each panel. VOs includes cortical alveoli oocytes.

**Figure 2:**
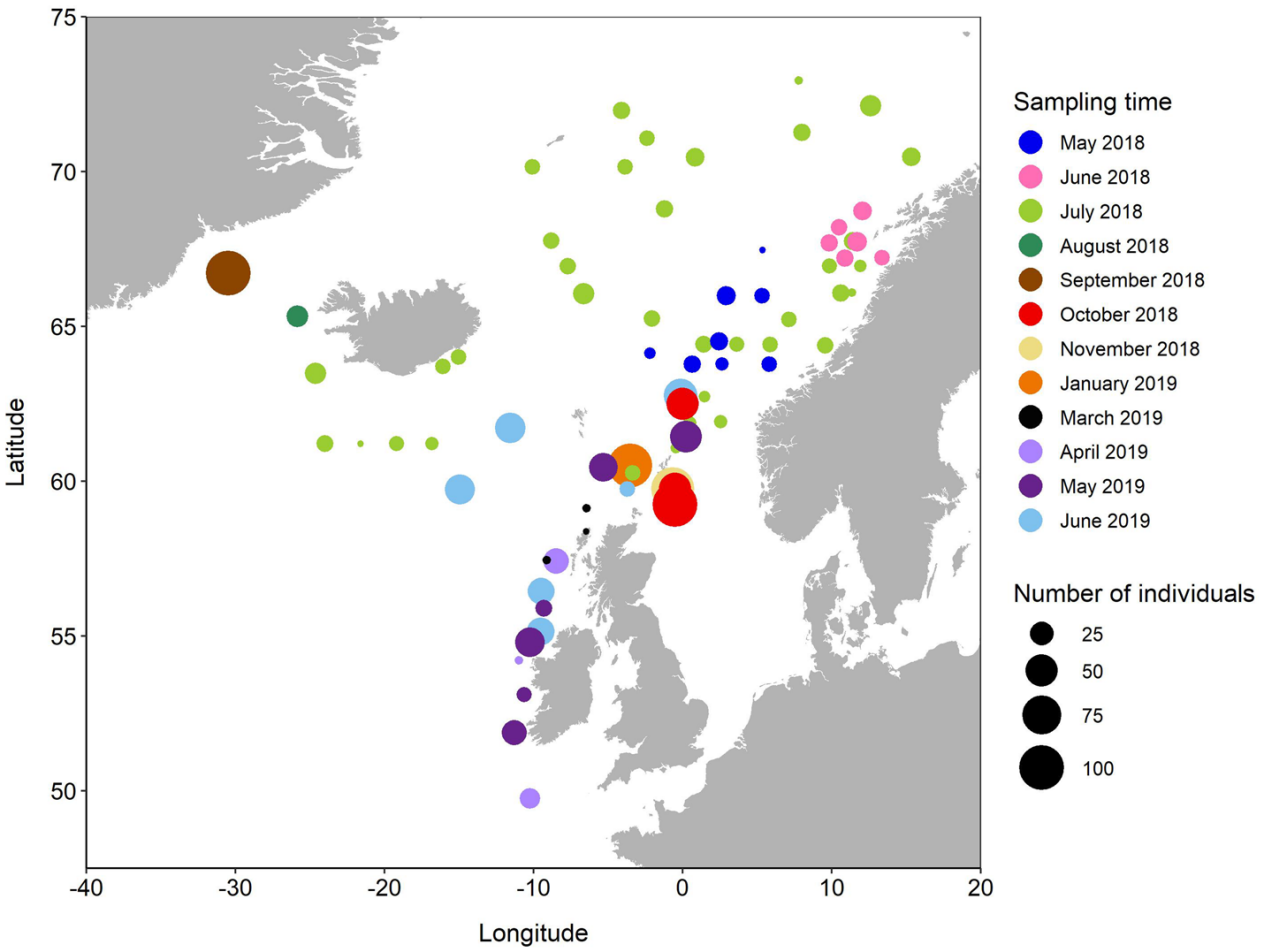
Map with location and number of all mackerel female samples collected from May 2018 to June 2019. The map was created using R v4.0.4 (https://www.r-project.org/) (see details at “Material and methods” section).

Addressing firstly “the population (wholemount) data set” of 1561 individuals (Table S2), the relative frequency of early-spawning (ORC1), mid-spawning (ORC2), and late-spawning (ORC3) females changed significantly as the spawning season progressed, although with dissimilarity between 2018 and 2019 (Supplementary, Fig. S4). Overall, a significant difference was found among the ORCs frequencies between the two field-sampling years (two-way ANOVA; *p* = 0.003). In June 2018, over 60% of the females caught were very late spawners or spent (ORC4), this relative frequency increased to almost 90% in July 2018 (Supplementary, Fig. S4A). For 2019, the ORC4 in June was about 50% (Supplementary, Fig. S4B). Combining these 2018 and 2019 data sets, the subsequent comparison showed that July 2018 clearly differed in terms of ORC (a posteriori Tukey test; Supplementary, Fig. S5). More females in mid-spawning were recorded in May and June 2019 compared to the same months in 2018, though this noted difference was statistically insignificant (Supplementary, Fig. S5). Altogether, these outlined variations in ORC (Fig. 1) may be related to survey coverage, i.e. in 2018 these samples were collected in Nordic waters, while in 2019 exclusively within the main spawning area (Fig. 2).

**Figure 3:**
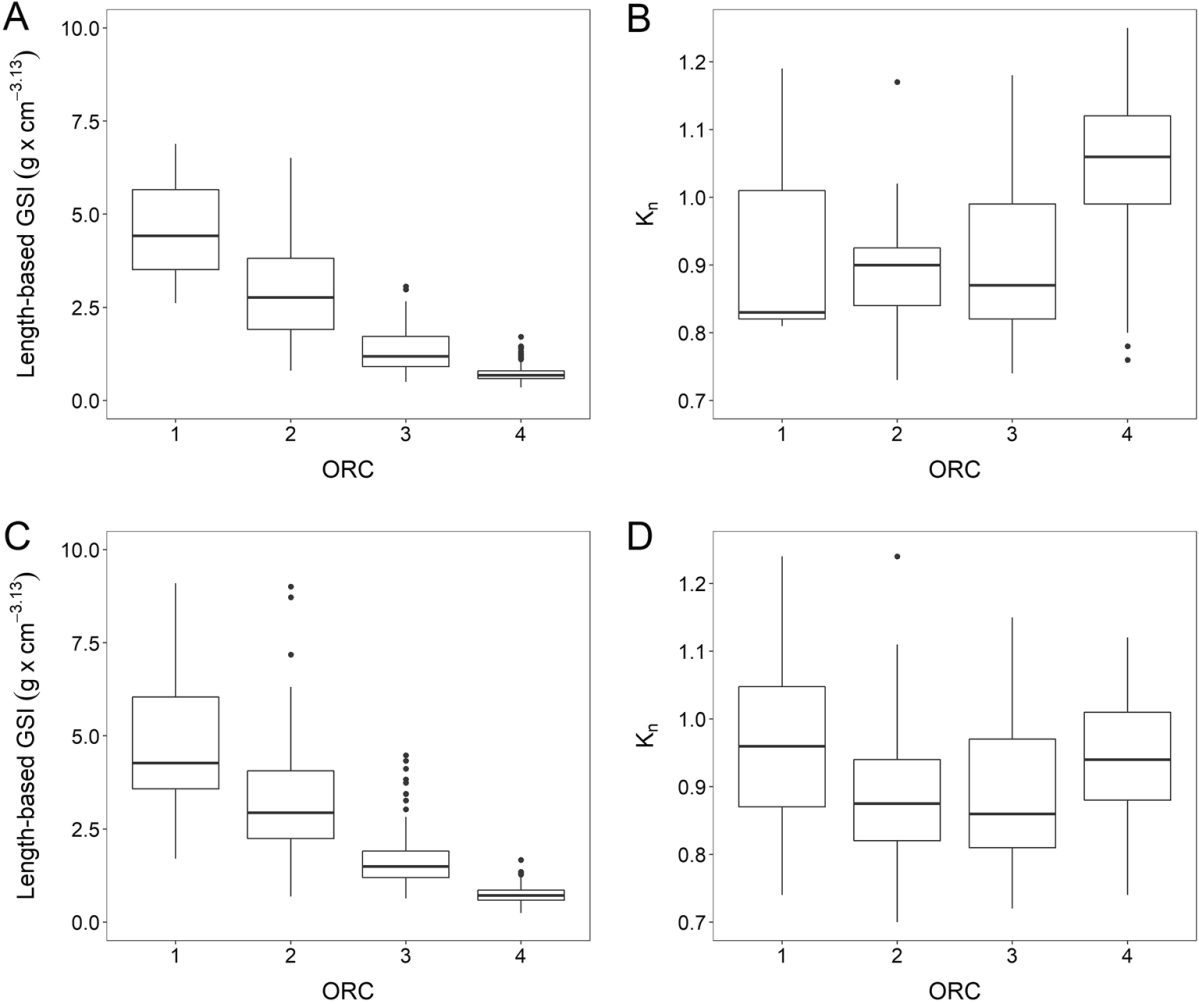
Length-based gonadosomatic index (GSI_TL_) (**A** and **C**) and relative condition (K_n_) (**B** and **D**) as a function of "stage of spawning", represented by ORC. Top panels (**A** and **B**) represent samples collected and analysed in wholemount in 2018, and bottom panels (**C** and **D**) in 2019. For each box plot, the thick line is the median value, top and bottom lines indicate the 75th and 25th percentiles, respectively, and whiskers indicate maximum and minimum value.

Population-level ORC and biometrics appeared linked, the latter represented either by total length (TL)-based gonadosomatic index (GSI_TL_) or relative condition (K_n_) (Fig. 3). The 2018 results showed that K_n_ was higher (*p* < 0.001) in late- (ORC3) and very late-spawning and spent fish (ORC4) compared to early-spawning (ORC1) and mid-spawning fish (ORC2) (Fig. 3B). In 2019, K_n_ values were more similar across ORC but highest (*p* < 0.001) in ORC1 and ORC4 (Fig. 3D). However, once more, the resulting significant differences in K_n_ between spawning status and years (two-way ANOVA, *p* < 0.001) might be due to survey coverage and time: mackerel females sampled in 2018 had most likely started feeding^26^ since they were mainly collected in the Norwegian Sea (Fig. 2).

K_n_ values restricted to the 144 OPD samples (Supplementary, Fig. S6B, D) showed very much the same trend with ORC as just presented above for the population data set (Fig. 3B, D; Supplementary, Table S2), except for some situations where OPD-related K_n_ values were slightly higher, as for example ORC1 in 2018 (Supplementary, Fig. S6B) and ORC3 in 2019 (Supplementary, Fig. S6D). This illustration speaks for that the OPD samples were a representative subset, at least in these regards, see also the related weight-at-total length [W-at-TL] plot (Supplementary, Fig. S7).

### Presence of postovulatory follicles and atresia

Both POFs and various degrees of vitellogenic atresia (Eα and Lα) were frequently annotated within the spawning season. For POFs, the corresponding volume fraction (Vν_i_) showed an increase towards summer (Supplementary, Fig. S8A). However, a more pronounced presence of POFs was recorded in 2019 than in 2018, i.e., when the samples were collected within spawning areas (see above). Although atretic vitellogenic oocytes were detected in almost all months with samples taken, except in October, their presence was exceedingly low well off the spawning season (Supplementary, Fig. S8B). Thus, Eα atresia Vν_i_ peaked in July 2018, when mackerel most likely had ceased spawning and were feeding in the Norwegian Sea^26^ (see above). Higher levels of Vν_i_ were generally observed for late (Lα) than for early (Eα) atresia, the former also being much more persistent; all examples of atresia outside the spawning season referred to this stage (Supplementary, Fig. S8B). Focusing on patterns within the spawning period as such, i.e., consulting ORCs, we observed that the Vν_i_ of both a atresia stages increased as spawning progressed from ORC1 to 3, seeing thereafter, at ORC4, a collective drop in Eα atresia but for Lα atresia this drop being restricted to 2019 (Supplementary, Fig. S9). No clear difference in K_n_ was seen between fish with or without α atresia, neither any evidence of a K_n_ effect across different TL on the presence of a atresia (Supplementary, Fig. S10).

PVO atresia was detected during the resting and early maturation period (August–April; Supplementary, Fig. S8B). September 2018 was an extreme case with PVO atresia being observed in all individuals analysed for OPD, amounting to a mean Vν_i_ of 8% (Supplementary, Figs. S8B, S10), despite that this month showed the highest mean K_n_ within the 2018 study year (Supplementary, Figs. S10, S11). K_n_ was typically around and above 1.0 in individuals where PVO atresia was observed (Supplementary, Fig. S10).

### Oocyte diameter

Generally, phase-specific oocyte size represented by mean volume-based oocyte diameter (cODν_i_) (where c stands for “corrected to formalin-preserved diameter”) varied little over the year. PVO2 and PVO3 showed indications of being smaller in macroscopically staged early-maturation months (October to January) compared to corresponding spawning and post-spawning months (March to September) (Supplementary, Fig. S2A). A partly different pattern was observed for late PVO phases; PVO4a was seemingly largest in October, whereas PVO4b and PVO4c in January 2019 (Supplementary, Fig. S2B). Likewise, no clear temporal pattern in size was recorded for phase_i_-specific VOs (one-way ANOVA, *p* = 0.184 [VO1]—0.681 [VO3]) (Supplementary, Fig. S2C). However, cODν_i_ of FOM (germinal vesicle migration [GVM] and germinal vesicle breakdown [GVBD]) decreased along the spawning period (see below), except for GVBD in May to July 2018 (Supplementary, Fig. S2D). This high variation in July 2018 was due to one female in early GVBD and another female in late GVBD.

**Figure 4:**
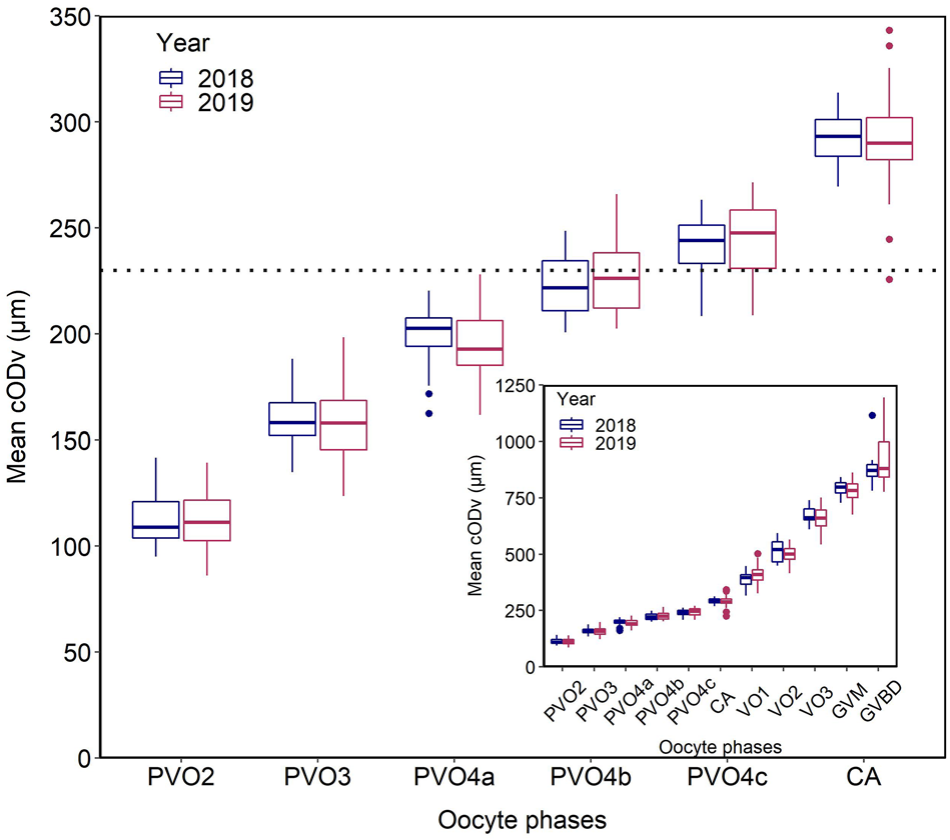
Mean oocyte size (cODν) by phase (_i_) during the spawning season (March to July). The insert shows the mean cODν for all oocyte phases, whereas the main plot presents a “zoom-in” on PVO2-4c and CA.

Concentrating on the spawning period, mean cODν_i_ increased in a smooth, likely slightly concave way from PVO2 to PVO4c (Fig. 4) but the growth in size picked up quickly from cortical alveoli (CA) to GVBD following an apparent linear trajectory up to GVM (Fig. 4, insert). Detailing the underlying phase-specific changes in mean cODν_i_ as a function of ORC, noticeable differences existed among and within PVOs and VOs (Supplementary, Figs. S12, S13). The mean cODν_i_ of PVO2-4a phases were rather stable whereas PVO4b-c oocytes displayed a more complex pattern (Supplementary, Fig. S12). Mean cODν_i_ at the CA phase was approximately similar before and during spawning (Supplementary, Fig. S13A) whilst oocytes in VO1 clearly became smaller as spawning progressed (though with one outlier; Supplementary, Fig. S13B). Mean cODν_i_ of VO2 and VO3 showed a rather mixed picture (Supplementary, Fig. S13C, D).

**Figure 5:**
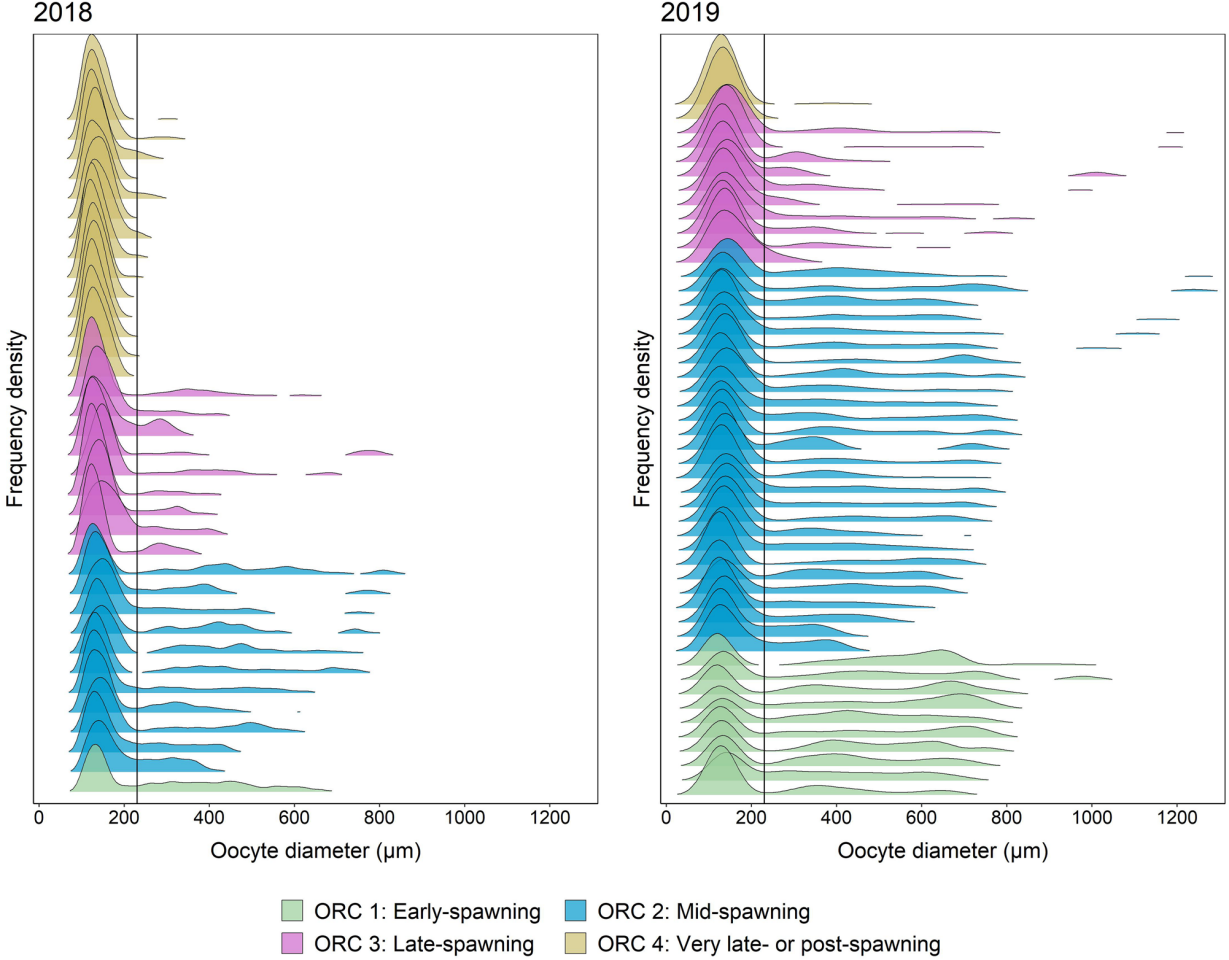
Smoothed oocyte size frequency distribution (OSFD) for each female used in the OPD analysis during the spawning period, according to oocyte ratio category. OSFD is organized in ascending order based on the maximum oocyte diameter within each ORC. The oocyte diameter for each female refer to the wholemount data analysis. The vertical line indicates the threshold value between PVOs and PVO4c (OD = 230 µm).

Switching to studying smoothed OSFDs across individuals (Fig. 5) (OD ≥ 100 µm), the frequency density of oocytes in the 230–800 µm range became gradually dampened as spawning progressed, see also weak indications of the same pattern following model smoothing restriction in OD to OD ≥ 230 µm (Fig. 4, Supplementary, Fig. S14). Spent ovaries (ORC4), except for six females, consisted only of PVOs (Fig. 5).

### Oocyte packing density and oocyte development

Mackerel showed a highly dynamic line of oocyte production throughout their reproductive cycle. Overall, the number of oocytes per gram of ovary (OPD) declined abruptly from PVO2 and PVO3 (millions g^−1^) via PVO4a–c (hundred thousand g^−1^) and, finally, to CA ending with GVBD (a few thousands or hundreds g^−1^) (Supplementary, Fig. S15). The phases PVO2, PVO3 and, PVO4a were always present in high densities (Supplementary, Fig. S15A, B). Their density showed a dome-shaped pattern, increasing from May to October 2018 (PVO2-3) and to November 2018 (PVO4a), then declining until the end of the forthcoming spawning period (Supplementary, Fig. S15A, B). Oocytes in PVO4b-c phases were also omnipresent, however, the number increased from August until November, i.e. during the macroscopic “resting period” (Supplementary, Fig. S15B). The number of PVO4b tended to be higher than PVO4c during most of the study period (Supplementary, Fig. S15B). The onset of maturation took place in October by the appearance of CA (Supplementary, Fig. S15C). The number of CA continued increasing from October until January, then decreased. Early vitellogenesis started in November, when primary vitellogenic oocytes (VO1) were noticed (Supplementary, Fig. S15C). Similarly to CA, the number of VO1 showed a dome-shaped pattern from October to June (Supplementary, Fig. S15C). The high variation recorded in several oocytes phases in March might be attributed to the lower number of individuals in different phases (n = 5) (Supplementary, Table S2). Mackerel was spawning capable (SC), in our study area, from March until July, when late vitellogenic oocytes (VO2-3) and FOM (GVM + GVBD) phases were present (Supplementary, Fig. S15D, Table S1). At least for 2019, the density of GVM and GVBD oocytes were inversely related, i.e. GVM oocytes declined in number when GVBD increased in number (Supplementary, Fig. S15D). The average OPD of GVM and GVDB was 900 and 460 oocytes g^−1^, respectively (Supplementary, Fig. S15D).

### Relative fecundity

**Figure 6:**
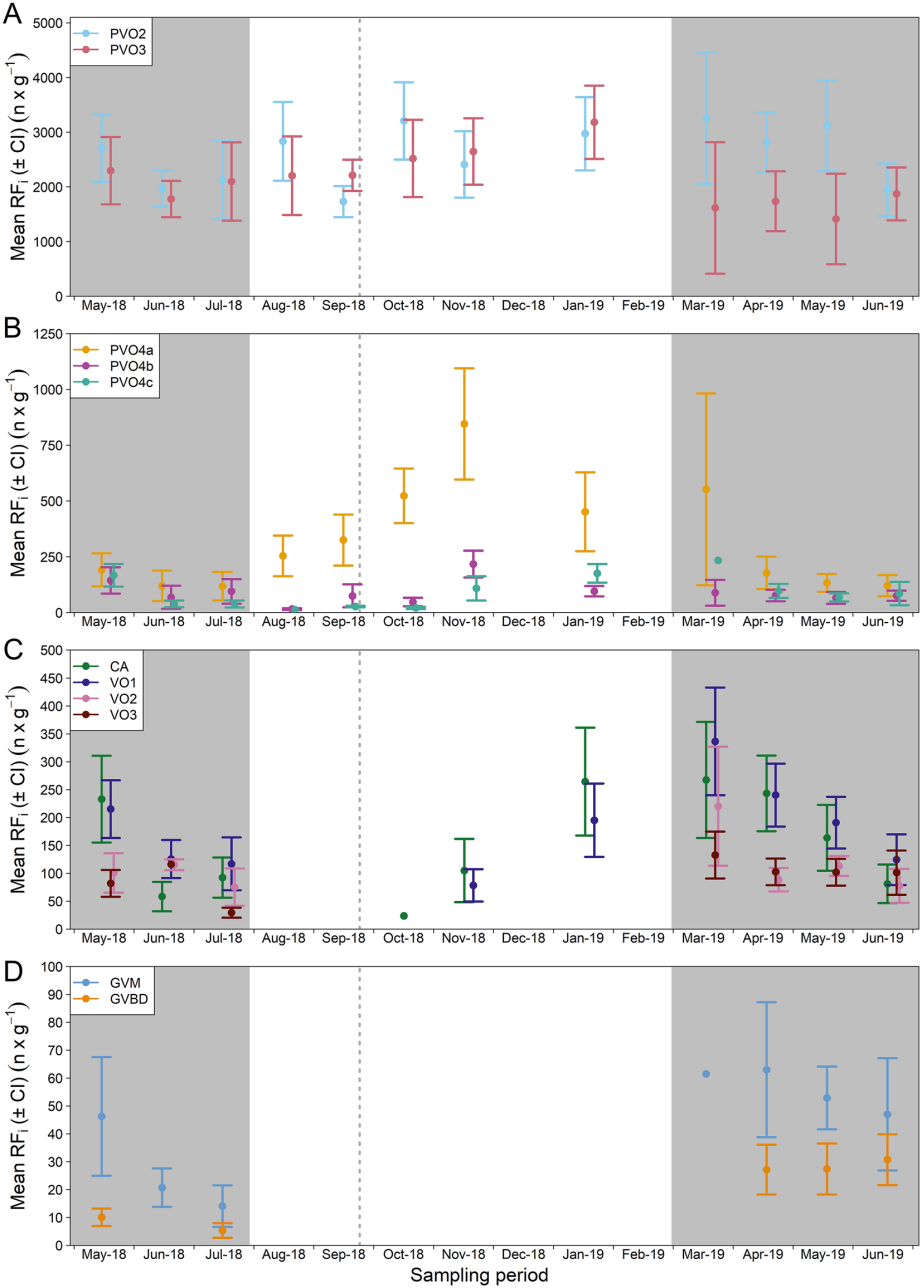
Monthly variation (mean and ± 95% confidence interval) in phase_i_-specific oocyte relative fecundity (RF_i_). (**A**) Monthly variation in RF_i_ of small previtellogenic oocytes (PVO2 and PVO3); (**B**) large previtellogenic oocytes (PVO4a–c), (**C**) cortical alveoli oocytes (CA), vitellogenic oocytes (VO1-3), and (**D**) oocytes in final maturation (germinal vesicle migration [GVM] and germinal vesicle breakdown [GVBD]). The autumn equinox is indicated (vertical dashed line). Grey bands indicate the spawning season. Note that the y-axis scale differs between panels. No samples were collected in December-18 and February-19.

From a more general perspective, temporal patterns in relative fecundity (RF_i_) estimates mirrored those for OPD_i_, simply because the former is given by multiplying the latter with ovary size (see below). Hence, mean RF_i_ (i.e. number of oocytes g^−1^ body weight) showed a similar seasonal trend as OPD_i_ for almost all oocytes phases, except for PVO2 and PVO3 which presented a rather flat RF_i_ over the sampling period (Fig. 6A). Overall, the mean figure for these two early PVO phases was > 2000 oocytes g^−1^ body weight (Fig. 6A). Mean RF_i_ for later PVO phases (PVO4a–c) was typically < 250 oocytes per g^−1^ body weight (Fig. 6B). Regarding CA to VO3, a dome-shaped pattern was found from the resting to end-of-spawning period (October 2018 to June 2019) (Fig. 6C). RF_i_ of FOM (GVM and GVBD) appeared at a higher level in 2019 than in 2018 (Fig. 6D). Trends in total length-based relative fecundity (RF_TLi_) compared well with those just outlined for RF_i_ (Supplementary, Fig. S16), considering here TL_j_ to be a more resilient body size biometric than W_j_ ((whole) body weight).

Setting the lower threshold value for PVOs at 230 µm, i.e. at PVO4c (Supplementary, Table S1)—the current result (see above and below)—instead of traditionally 185 µm^18^, i.e. at PVO4a (Supplementary, Table S1)—during the enumeration work significantly (paired t-test, *p* < 0.001) affected the resulting, aggregated RF_i_ (Supplementary, Fig. S17). Note here that a few females contained only PVO4a (at ORC4) and thereby dropped out in this pairwise comparation (ORC1-3). The corresponding reduction from RF_PVO4a-GVBD_ to RF_PVO4c-GVBD_ was around 29 and 20% for 2018 and 2019, respectively. Mackerel obviously ate during the spawning season, seen by no obvious drop in K_n_ with ORC (Supplementary, Fig. S17), a topic explored further on above.

### Quantifying de novo oocyte recruitment

**Figure 7:**
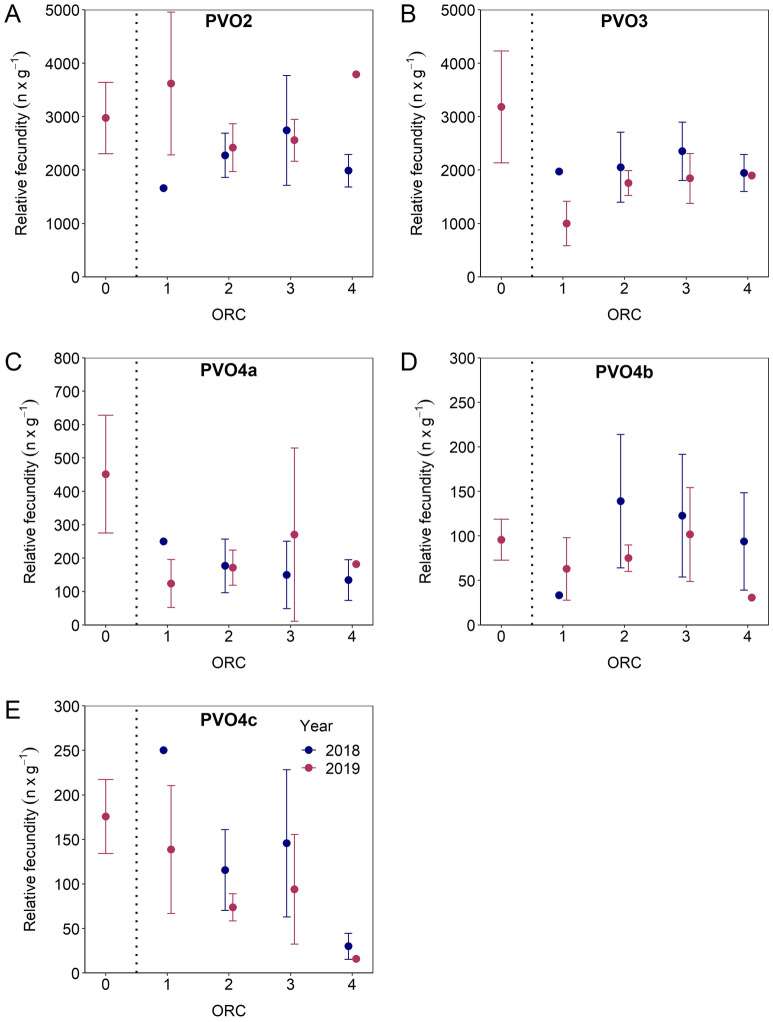
Mean (± 95% confidence interval) relative fecundity of previtellogenic oocytes in phases PVO2 to PVO4a–c by oocyte ratio category (ORC) split by 2018 and 2019. Vertical line separates prespawning individuals (ORC0) from those that have already started spawning (ORC1 to 4). Note that the y-axis scale differs between panels.

**Figure 8:**
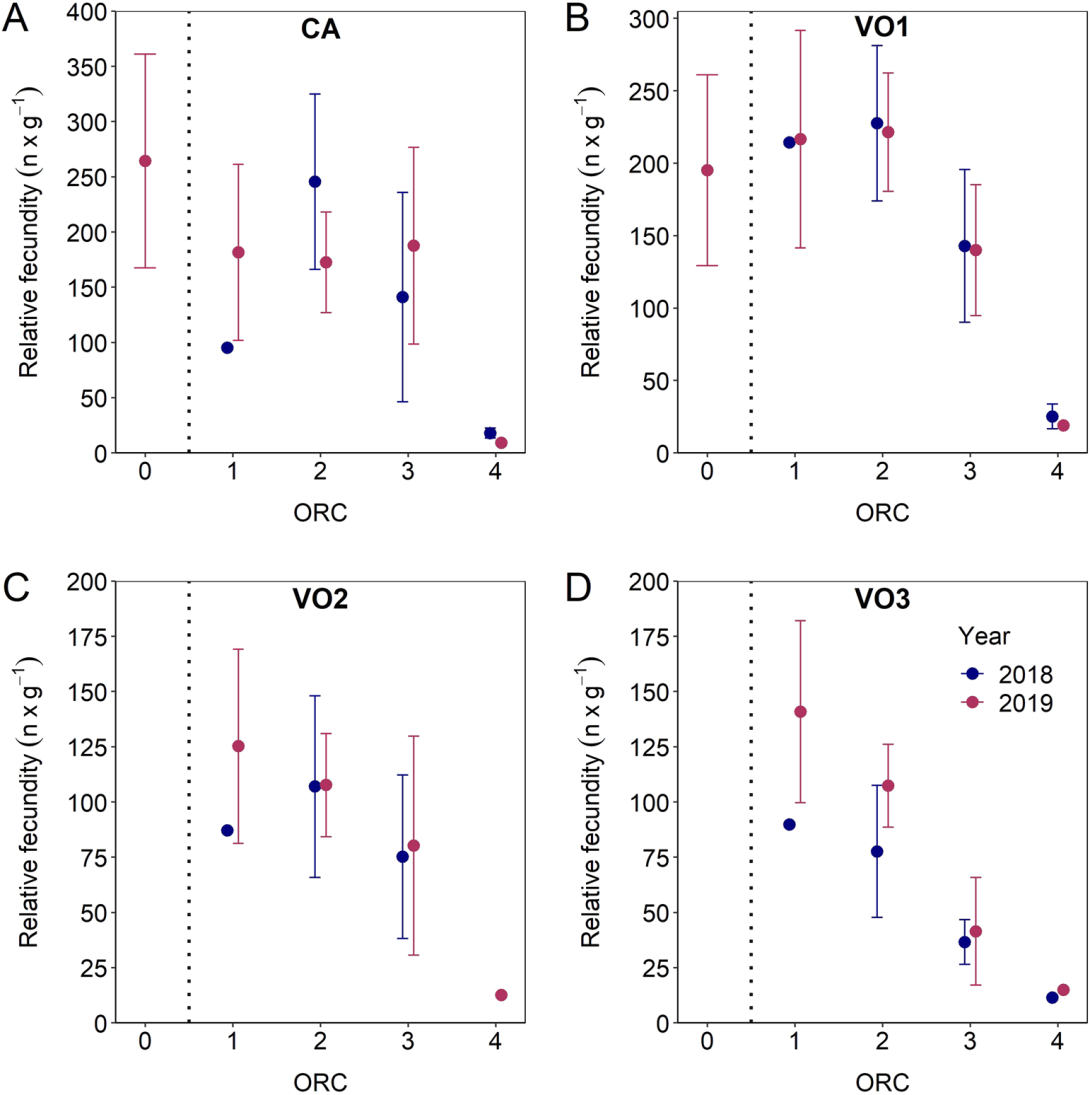
Mean (± 95% confidence interval) relative fecundity of cortical alveoli oocytes (CA) and vitellogenic oocytes (VO1-3) by oocyte ratio category (ORC) split by 2018 and 2019. Vertical line separates prespawning individuals (ORC0) from those that have already started spawning (ORC1 to 4). Note that the y-axis scale differs between panels.

**Figure 9:**
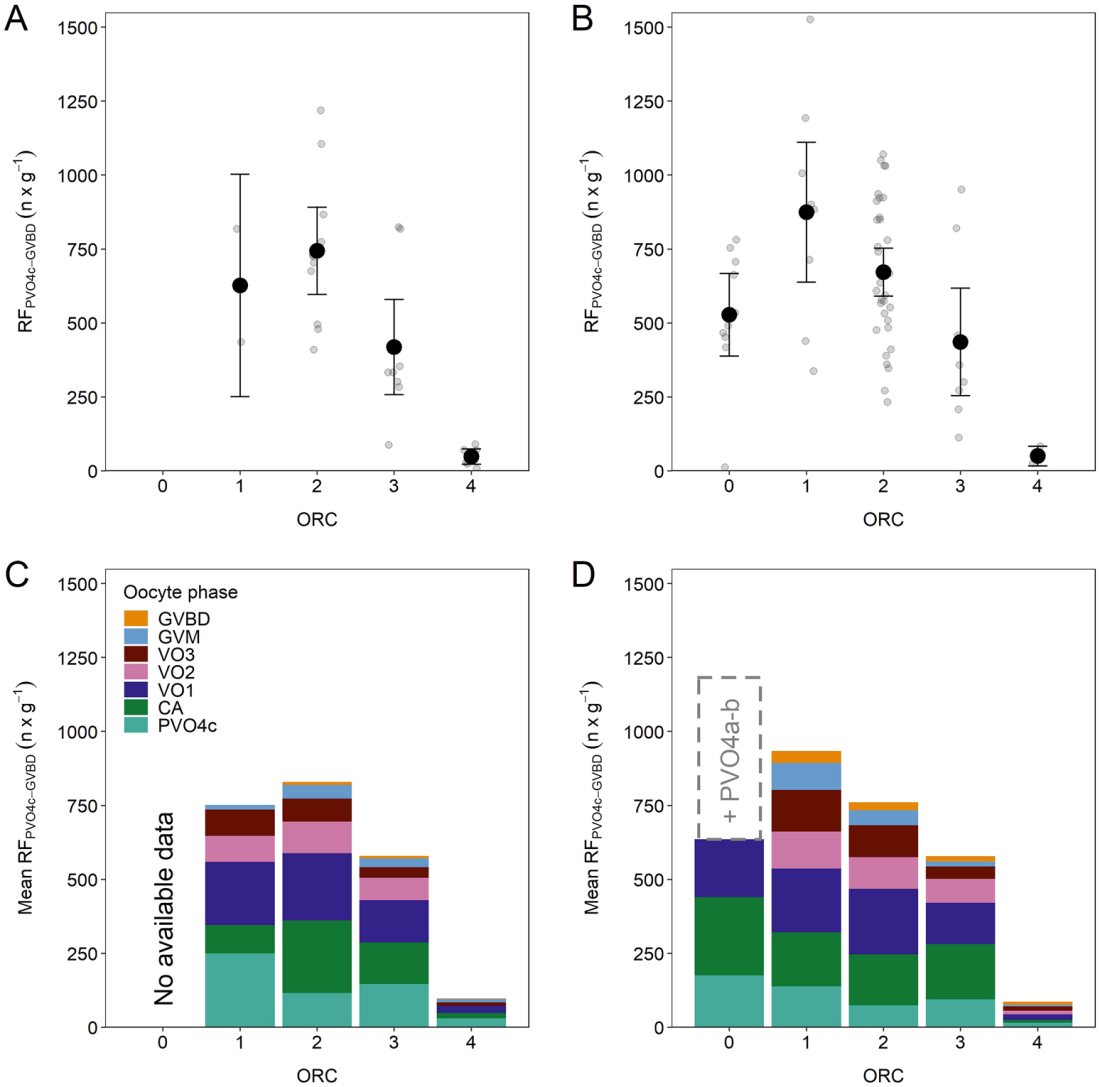
Mean (± 95% confidence interval) RF_i_ (relative fecundity) at PVO4c to GVBD in 2018 (**A**) and 2019 (**B**), and the sum of grand mean RF_i_ at PVO4c to GVBD in 2018 (**C**) and 2019 (**D**), split in all cases by oocyte ratio category (ORC). Grey circles in (**A**) and (**B**) represent the span in individual RF_i_. For (**C**) and (**D**) the contribution of each oocyte phase is detailed within the histogram. Dashed column in (**D**) indicates the RF_i_ when PVO4a and PVO4b are added to the fecundity estimate.

Successive estimates of relative fecundity (RF_ij_) from PVO2 to VO3 evidenced that the spawning period (ORC1–4) is a time of most active transfer of one type of oocytes to the next type of oocytes, but not equally applicable to all oocyte phases. There was a sign of an initial decline in respective RF_i_ PVO2-4b, i.e. from prespawning (ORC0) to early-spawning (ORC1), but then levelling off, from ORC1 to ORC4 (very late- or post-spawning) (Fig. 7A–D). Importantly, this description did not apply to RF_i_ of PVO4c which showed a pronounced decline with ORC (Fig. 7E), with the subsequent RF_i_ of CA presenting a resembling, but more chaotic picture (Fig. 8A). RF_i_ of VO1 followed a right-skewed dome-shaped trend vs. ORC (Fig. 8B), whereas RF_i_s of VO2 and VO3 were more in line with an on-going fall with ORC (Fig. 8C, D). At the assumingly representative, aggregated level (PVO4c–GVBD), RF_i_ exhibited a dome-shaped pattern throughout spawning, though with large individual variation at a given ORC (Fig. 9). So, de novo oocyte recruitment is evidently important in mackerel, exemplified foremost for 2019 where the RF_i_ in question increased by almost 65% between ORC0 and ORC1 (Fig. 9B). For 2018, RF_i_ increased from ORC1 to ORC2, but for this year missing ORC0 data excluded the possibility to track any initial RF_i_ change (Fig. 9A). For a so-called “standard individual”, i.e. summing the grand mean of phase_i_-specific RF_i_, a higher final mean RF_i_ was registered; compared to the estimates just presented (Fig. 9A, B), the difference ranged from 7% at ORC1 in 2019 up to 102% at ORC4 in 2018 (Fig. 9C, D).

### Number of batches

Considering the seemingly representative, prespawning mean RF_i_ in 2019 (ORC0, 528 oocytes body g^−1^) (Fig. 9B) plus PVO4c de novo recruiting (160 oocytes body g^−1^—found by subtracting ORC4 figures from ORC0 figures) (Fig. 7E), i.e. totally 688 oocytes body g^−1^, divided by batch fecundity (40 oocytes by body g^−1^)^27^, the typical mackerel female apparently produced ~ 17 batches in this particular year. Consulting instead the grand mean batch fecundity reported by ICES^18^ (~ 30 and 34 oocytes by body g^−1^ in 2016 and 2019, respectively), the number of batches increases to ~ 20–23. So, this number on batches released per individual is strongly dependent upon the batch fecundity, an issue not pursued further here.

## Discussion

Studies within applied fish reproductive biology have mostly targeted advanced developing oocytes, i.e. primarily VOs or FOM/HO (hydrated oocytes), when aiming at studying temporal and/or spatial fecundity dynamics^9,13,14^. However, over the recent years, several studies have pointed at the importance of focusing on the actual PVO production as well to better understand oocyte recruitment processes and thereby the underlying fecundity formation as such^10,13,21,24,25,28,29^. In the stock assessment of mackerel, one of the biggest problem up today has been to unveil the actual fecundity type and thereby run the most adequate EPM^20,30^ to provide the SSB index possibly nearest to the real SSB^18,20^. We combined the auto-diametric^31,32^, OPD^15^, and ultrametric method^10^ to precisely and accurately quantify this species’ oocyte recruitment dynamics. To our knowledge, no earlier study has been able to present such an in-depth quantitative insight in the fecundity type of any teleost, following standardization by the “stage of spawning” (ORC). As addressed below, the fate of PVO4c oocytes is a central issue. From our perspective, one of the most key results in this article is the ability to estimate the numerical decline during spawning of this final PVO phase as this figure should represent those PVOs subsequently turning into VOs and ultimately pelagic eggs, following adjustment for any atresia. In other words, such detailed information clarifies the degree of de novo oocyte recruitment. In practical terms, one basically only needs to sample prespawning (ORC0) as well as spent individuals (ORC4) to get this strength of oocyte influx. Sampling only ORC0-females seems, however, an incomplete protocol because we found that RF_i_ PVO4c does not necessarily goes down to zero at ORC4.

### Spawning progress

The ultrametric method, as described by the authors^10^, was “designed for determinate spawners, but might be extended to include indeterminate spawners”. Our following-up analysis confirmed that this novel, wholemount-automated technique can be applied on indeterminate species—presently validated by tracking GSI_TL_—and thereby be a most useful supplemental tool to determine the fecundity type of many other fish species as well, provided they are serial spawners. From a theoretical perspective, this wider prospect of applicability rests on the assumption that ΣPVO is a stable reference point (numerator) (whereas Σ(VO + FOM) (denominator) varies)^10^. For indeterminate spawners the constancy in ΣPVO is, however, challenged by recruiting PVO4c oocytes, i.e. a drop in ΣPVO. Consulting OPD results, this effect is trivial; cf. the exceedingly high OPD ΣPVO2-4b compared to OPD PVO4c. However, the ultrametric method utilises automated image analyses (Supplementary, Fig. S3), where the ΣPVO is significantly lower (Fig. 1), apparently because many PVOs are “hidden”, e.g. around or under the large VOs. Thus, further advancement in this technique might be required, but we believe that its role served the purpose, supported by the above-mentioned positive validation, speaking for that there is a comparable error in both the numerator and denominator in the outlined formula. During this image analysis, we learned that cytoplasmic structures like the circumnuclear ring (CNR) might be detected in unstained wholemount preparations (Supplementary, Fig. S3), but any operational use requires further tests, e.g. presumed PVO4c might in cases turn out to be PVO4b.

### Oocyte recruitment

PVO dynamics of mackerel mimics the one seen in another teleost warm-temperate, serial spawner, the European hake (*Merluccius merluccius*)^25^. Small oocytes, represented by phases PVO2 to PVO4b, were omnipresent constituting thereby “the reservoir of oocytes”^9^. However, our results contrasted in these respects with the finding of Greer-Walker et al.^4^ that the number of PVOs decline from October to January, then increase until June, indicating a typical feature of a determinate fecundity type. In fact, we observed the opposite pattern; PVOs per body gram increased past spawning to early maturation, i.e. from May to October–November but then declined during spawning. More specifically, the number of PVO4c apparently peaked during the resting period, whereas the following CA started to appear in October^4^, i.e., a few weeks after autumn equinox, in other words, when the night starts to become longer than the day.

Serrat et al.^25^ indicated for European hake that oocytes in PVO4b, and subsequently PVO4c, were recruited to the vitellogenic pool during the spawning season based on OPD results regressed on visual maturity stage classification. Instead, we used the ORC system to detect precisely any signal of such de novo recruitment during spawning. Notably, our results on relative fecundity showed that oocytes in phase PVO4c become significantly fewer as spawning progresses, reflecting that mackerel is a true indeterminate spawner^3^, principally because this numerical fall cannot be attributed to intensified PVO atresia (see below). The following recruitment to subsequent oocyte phases can be tracked by an increase in relative fecundity of VO1 from ORC0 to ORC2, before fading away and seeing a sharp decline in VO1, VO2, and VO3 as successive egg batches are produced and released. The relative fecundity of CA, presenting the transition from PVOs to VOs, showed a complex picture between ORC0 and ORC2 but also in this case a subsequent fall was evidently in place.

Presence or lack of hiatus between PVOs and VOs have commonly been used to indicate whether de novo oocyte recruitment is in place or not, and thereby indeterminacy or determinacy, respectively. However, the presence of hiatus is not an exclusive feature in indeterminate spawners species, especially when samples are collected prior to spawning^8^. As an example, indications of de novo oocyte recruitment were recently found in spawning cod^10^, a classical determinate spawner species^5^. Based on the above-mentioned controversy about mackerel fecundity type, including any presence or lack of a hiatus between PVOs and VOs, we currently revised this topic. Our statistically-established, wholemount 192 µm threshold value between PVOs and CA-VOs is, when considering any method uncertainty, in practical terms fully comparable to the one reported and used earlier^4,32^, namely 185 µm. However, our in-depth histological (stereological) analysis showed that these oocytes are highly likely recruited from 230 µm onwards (using consistently formalin-fixed diameter as reference), i.e. when the oocytes are in the PVO4c phase. In the mackerel stock assessment, the 185 µm value has been adopted as the lower size threshold in fecundity calculations^30^. In other words, possibly some PVO3 but certainly PVO4a and PVO4b oocytes are also included in the presented ICES fecundity estimate (cf. Fig. 9D; Supplementary, Fig. S3 and Table S1). Rightly so, some of this numerical overestimation might be counter-balanced by atresia loss correction^30^, but to reiterate, PVO atresia during spawning seems not to be an issue. We therefore advocate that the protocol established as a part of this article should be considered as a supplement to the historical fecundity protocol^30^, since the current one exhibits phase-specific oocyte numbers and thereby provides a wealth of insight in fundamental fecundity formation (as well as can be applied on any species regardless of fecundity type, if this being of interest). Anyway, the lower diameter threshold when undertaking mackerel fecundity estimates (except for batch fecundity) should, from our perspective, be increased from 185 to 230 µm. The latter value also falls much better in line with e.g. 240 µm in Atlantic herring (*Clupea harengus*)^33^ and 250 µm in Atlantic cod^21^. Putting these considerations aside, the main advantage of the science outlined here is that the addition of the ultrametric method makes it possible to study the temporal production of oocytes recruiting instead of adopting a static lower diameter threshold.

### Atresia

Reabsorption of vitellogenic oocytes at the end of a spawning season—the “mopping up” process—is a characteristic^34,35^ but not necessarily an obligatory feature^11^ of indeterminate species. Furthermore, skipping spawning is a common trait in iteroparous fish, even after vitellogenesis has started, due to a series of influential factors^36^. In the case of mackerel, which we here clearly show is an indeterminate spawner, atresia was detected in almost all months presently analysed. The highest incidence of atresia was detected in the end of spawning, including for the germinal vesicle migration phase. The atretic pattern suggests that mackerel may abort the production of the remaining egg batches and instead focus on feeding more actively, or that the environmental conditions might be unsuitable for further spawning^36^. After spawning mackerel migrate into the Nordic Sea for active feeding and stay there until overwintering^26^. At the end of spawning season, we observed mackerel with lower body condition, however, no differences were recorded in body condition between females with and without alpha (vitellogenic) atresia. In Chilean jack mackerel (*Trachurus murphyi*), for instance, poor body condition results in heightened atretic intensity (in those individuals with atresia) but not atretic prevalence (when referring to the whole population as such)^37^. Overall, the presence of atresia seems to affect just the final individual fecundity output, i.e. females with atretic oocytes apparently will continue spawning, however, producing less eggs than those females without atresia^38^. PVO atresia^39^ was presently recorded in mackerel for the first time, with no relationship with body condition. Therefore, the presence of PVO atresia might relate to that these oocytes were in a too advanced developed phase for the period.

## Conclusion

Our data on mackerel suggest that the so-called “lines of evidence” used as conceptual criteria to distinguish between determinate and indeterminate spawners^4,17^ should be revisited as they, although informative, have not been sufficient enough to classify the mackerel into the right fecundity type. The combination of novel methods—OPD theory^15^ and ultrametric method^10^—allowed us to document that mackerel indeed has an indeterminate fecundity based on de novo oocyte recruitment recorded within the spawning period. Any sign of a hiatus between PVO4c and CA-VO was not an intrinsic part of this conclusion, as the presence of a hiatus or not can be a highly unreliable criterion^10^. Also, this eventual gap in diameter starts to happen at a time in oogenesis when oocytes incorporate cortical alveoli, thus probably speaking for a fast diameter increase when these glycoproteins appear in the blood stream^34^ and thereby in oocytic follicular capillaries. Although both CA and VO1 showed examples of pulsed production during spawning, our conclusion on indeterminacy is primarily based on the associated clear fall in RF_i_ PVO4c. Such a negative trend cannot be explained by heightened PVO atresia as this type of atresia happens outside the spawning season. Revealing this dynamic requires most sophisticated tools, as the present ones. We would argue that even though our method approach is surely complex (combining histology/stereology and principal physics) and partly highly technical (e.g. accounting for shrinkage and applying advanced image analysis), the extensive work effort involved pays off. The issue of de novo recruitment has been discussed for decades within fish biology but this is the first time when a reliable, quantitative estimate is presented and then for one of the commercially most important stocks in Europe, the mackerel, where such information is not only of basic but also applied interest.

It is our hope that this method investigation will launch increased attention to the definition of the lower oocyte size threshold value to include in fecundity estimates within the AEPM. Our analysis shows that this threshold in mackerel is clearly not 185 µm, but we cannot firmly say that it is 230 µm, or, e.g. 240 µm. This caution refers to that the threshold is the consequence of histology (where the section angle might vary) and following mathematical correction to formalin-fixed value. Any important deviation from reproductive trait reality—cf. 185 vs. revised 230 µm—is, however, shown to markedly influence RF_i_, which undoubtedly will translate into biases in the SSB index. Rather than presently going into this overall AEPM assessment, our results speak for that the corresponding historic time series should be revisited keeping in mind that the long-term trend as such is of special interest^18^. Logically, if/when such a recalibration program is initiated, another key issue would be to pinpoint how annual variation in mackerel body condition impacts its reproductive output^26^, e.g. RF_i_ as a function of K_n_.

## Methods

### Ethics approval

No further specific permissions for the below-outlined sampling scheme during research surveys were required as all mackerel were obtained in full accordance with international guidelines and standards through the International Council for the Exploration of the Sea (ICES), currently consisting of 20 member states (www.ices.dk). Regarding mackerel samples from landings, fishing rights were attributed to each commercial vessel. All specimens studied in this article were deceased in line with standard practise when the present analytic work commenced.

### Samples collection

The ovarian development of the mackerel was traced from May 2018 to June 2019, except in December 2018 and February 2019, along the Northeast Atlantic Ocean (Fig. 2, Supplementary, Table S2). The corresponding adult samples were provided from research surveys and fish landings, covering the feeding and overwintering periods when schools tend to agglomerate, but also during the spawning season when mackerel are more dispersed^40^. Totally, 1583 female mackerel were processed for subsequent analysis (Supplementary, Table S2). Total length (TL; cm), whole body weight (W; in g), sex, and maturity stage were individually recorded. Ovaries were initially visually staged, either according to the Institute of Marine Research (IMR) maturity scale (8 stages; see below)^41^ or the Walsh maturity scale (6 stages)^42–44^. However, for the sake of consistency and as the majority of mackerel samples collected were initially classified based on the IMR standard maturity scale, we undertook a consistent reclassification using the scale of Mjanger et al.^41^, where 1–2: juveniles, 3–5: maturing, 6: spawning, 7: spent, 8: resting. All mackerel females in spent and resting stages were classified using Mjanger et al.^41^ maturity scale. Ovaries were preserved in 3.6% neutral buffered formaldehyde. Back in the laboratory, all formalin-preserved ovaries were carefully weighed (OW; 0.001 g).

### Fish metrics

The length–weight power function relationship (R^2^ = 0.785, *p* < 0.001) was calculated by combining all females caught, finding the slope value (b) to deviate from 3 (b = 3.13, 95% confidence interval (CI) = 0.08) (Supplementary, Fig. S7). Therefore, K_n_ was estimated rather than the traditional Fulton’s K (with b = 3) based on the ratio between individually observed weight and expected weight: K_n_ = W_observed_/W_expected_^45^. GSI_TL_ was also calculated, using the formula GSI_TL_ = 10^4^ × (OW/TL^3.13^)^33^.

### Wholemount and histology

A subsample in the mid-part of the ovary was removed for wholemount analysis (cf. the auto-diametric method^31^). This subsample was considered representative of the whole ovary as earlier tests have evidenced that the mackerel ovary is homogenous in structure^46^, as further supported in our histological work (see below and Supplementary, Fig. S18). Oocytes were dissociated by using an ultrasonic pen for around 10 s, stained in toluidine blue and washed multiple times in formalin solution to remove any surplus staining^10^. Oocytes were randomly spread out in a petri-dish and three distinct images were taken under the stereomicroscope to automatically measure (ImageJ, plug-in ObjectJ) oocyte diameter (OD) (see below). A 100 µm value was set as minimum analytic threshold value as some of the tiniest oocytes might have been lost during the washing procedure^10^. All images were inspected visually and irrelevant recordings, such as pieces of connective tissue, removed. A few (N = 22) out of all analysed individuals showed oocytes < 100 µm and were excluded (final wholemount N = 1561; Supplementary, Table S2). A total of 404 ovary samples (Supplementary, Table S2) were randomly selected for histology/stereology and prepared according to standard procedure using an ascending concentration of ethanol (70% up to 96%), embedded and mounted in Technovit® 7100, sections cut 4 µm apart and stained with 2% toluidine blue and 1% sodium tetraborat and, finally, scanned with a × 40 objective and a resolution of 220 nm/pixel (Hamamatsu S60).

### Oocyte size frequency distribution

Based on the above-outlined microscopic criteria (Table S1) (see also below), 230 µm was set as the maximum diameter for PVOs in the below oocyte ratio (OR) formula and following oocyte ratio category (ORC) classification. We realized that PVO4c might possibly be slightly higher (Supplementary, Table S1) but 230 µm was found to be a conservative figure in a series of pilot tests. Overall, the studied PVOs ranged currently from 100 to 230 µm (thus we did not include the tiniest PVOs, the PVO1 (Supplementary, Table S1), and the following CA, VO and final oocyte maturation (FOM) from 231 to 1100 µm, where the given lower (100 µm) and higher extreme (1100 µm) follow from method-defined restrictions (see above and below).

### Spawning progress

To study mackerel oocyte recruitment patterns during spawning, we calibrated “stage of spawning” wholemount criteria recently developed for cod^10^. More specifically, the OR (oocyte ratio) was used to reflect each mackerel female’s spawning status given by the number of PVOs divided by the total number of VOs and FOMs; OR = ΣPVO/Σ(VO + FOM)^10^. Hence, a relatively higher OR indicates further advancement in spawning (Table 1). Due to the foreseen absence of a clear hiatus between PVO and CA (or possibly VO)^4^, an average threshold value between these oocytes categories (see detailed definition at oocytes phases in subsection below) was firstly established, studying 28 random females collected during the two spawning seasons (May–July 2018; April–June 2019). The Gamma/Gaussian mixture method (“gmm”) was applied (R package: shazam^47^, and the density distribution function selected based on the data distribution^48^). Thereafter OR was individually estimated for all remaining females (2018: N = 529; 2019: N = 469) from this active part of the reproductive cycle (Fig. 1, Supplementary, Table S2). Note that hydrated oocytes were not measured; histology screening along with wholemount analysis showed that the follicle layer typically becomes detached at OD > 1150 µm. Therefore, OD = 1100 µm was set as the maximum diameter value in the OR calculation. More specifically, FOM was defined to include oocytes up to this size (Supplementary, Table S1). To make sure that the examined material also included samples from females in a prespawning status, playing the role as OR reference point, mackerel collected in January 2019 (N = 96) were also analysed (see below). In our study, the January 2019 samples represent the closest prespawning fish data we had. Finally, OR was grouped into OR categories (ORC) modified from Anderson et al.^10^: ORC0 = prespawning, ORC1 = early-spawning, ORC2 = mid-spawning, ORC3 = late-spawning, and ORC 4 = very late- or post-spawning (spent) (Table 1).

**Table 1:** Description of oocyte ratio category (ORC) and the oocyte range (OR) for Northeast Atlantic mackerel (*Scomber scombrus*) based on the description presented in Anderson et al.^10^, using wholemount analyses.

ORC	OR range	Description
0		Prespawning fish. Individuals in primary vitellogenesis (VO1), no spawning has taken place. Lower or rather equal number of PVOs compared to VOs
1	≤ 1	Early-spawning fish. Larger or rather similar proportion of VOs compared to PVOs. Hydrated oocytes are in general absent
2	> 1 and ≤ 3	Mid-spawning fish. Oocytes in final maturation (FOMs) are commonly noticed
3	> 3 and ≤ 15	Late-spawning fish. One or more VO cohorts present. PVO ratio becomes higher
4	> 15	Very late- or post-spawning fish. Presence of mostly PVOs, almost no VOs are seen

Note that especially previtellogenic oocytes (PVOs) might be underestimated compared to OPD-based results. For further information on number of oocytes recorded automatically by the image analyser, see Supplementary Fig. S1.

Before applying the ORC method, the realism was tested by studying the performance of total length-based gonadosomatic index (GSI_TL_) as a function ORC. Both the complete wholemount data set (N = 1561; Fig. 3A, C; Supplementary, Table S2) and the more limited OPD data set (N = 144; Supplementary, Fig. S6A, C) showed a gradual, marked decline in median GSI_TL_ with ORC (one-way ANOVA, *p* < 0.001). The separate trends within and across the 2 years were comparable (two-way ANOVA, *p* = 0.35 for wholemount data set, and *p* = 0.53 for OPD data set). Thus, we considered the ORC method to be successfully validated. Also, these results showed that individual GSI_TL_ could not replace the current ORC system as a “stage-of-spawning” metrics due to the relatively large individual variation in GSI_TL_ at a given ORC.

### Oocytes phases

Prior to any following numerical quantification using the OPD method (see below), relevant cells and structures in the ovary were annotated based on microscopic (histological) classification schemes. Oocytes were classified using a total of 13 categories (Supplementary, Table S1). Previtellogenic oocytes were subdivided into 6 phases [PVO1–PVO4a–c]^49^, and the following developing oocytes into 4 phases (cortical alveoli [CA], primary [VO1], secondary [VO2] and, tertiary vitellogenesis [VO3])^50,51^. Final oocyte maturation (FOM) phases were subclassified as germinal vesicle migration (GVM), germinal vesicle breakdown (GVBD) and, hydrating (HYD)^50,51^. Cortical alveoli oocytes, referring to a gonadotropin independent phase^34^, were grouped together with vitellogenic oocytes, as commonly done, e.g.^13,50–52^. Atretic cells (PVO atresia, and early (Eα) and late alpha (Lα) (vitellogenic) atresia) as well as postovulatory follicles (POFs) were identified (Supplementary, Table S1). Additional structures represented by ovarian wall and stoma, blood cells, oocyte follicle layers were also noticed^25^ but grouped as ‘others’. As late POFs and beta atresia are hard to separate microscopically^30,38^ these structures were added to the ‘others’ category.

### Oocyte packing density

The oocyte packing density (OPD) formula^15^ was applied on 144 random females (Supplementary, Table S2) to estimate the number of oocytes per gram of ovary in each oocyte phase (i) based on the refined OPD formula^25^:

$$OPD_{ij} = log\left[ {V_{Vij} \times \left( {\frac{1}{{\rho_{o} }}} \right) \times \left\{ {\frac{{\left( {1 + k_{ij} } \right)^{3} }}{{\left( {8 \times k_{ij} } \right)}}} \right\}} \right] + 12.28 - 3 \times log\left( {cOD_{vij} } \right)$$

where OPD_ij_ is the phase_i_-specific oocyte packing density by female (_j_); V_Vij_ is the volume fraction of phase_i_ oocytes by female (_j_); ρ_o_ is the specific gravity of the ovary; k_ij_ is the mean shape factor of phase_i_ oocytes by female (_j_); cODν_ij_ is the mean phase_i_ volume-based oocyte diameter by female (_j_) corrected for shrinkage^22^.

#### Volume fraction

V_Vij_ was determined based on the Delesse principle, where area fraction is equivalent to volume fraction^53^. “Hits” were counted by a Weibel Grid with 500 grid points (ImageJ; https://imagej.nih.gov/ij/ and ObjectJ; https://sils.fnwi.uva.nl/bcb/objectj/). Typically, 12 (5–18) samples were analysed per month (Supplementary, Table S2). The entire histological section of small and medium size ovaries (< 100 mm^2^) was considered (Supplementary, Fig. S18), whereas half of the section for large ovaries (≥ 100 mm^2^), i.e. those in the most advanced maturity stages. Pilot tests clarified that the number of hits for the tiniest PVOs (PVO1) were too low to qualify for further OPD_PVO1_ quantification and thereby excluded. Hence, the applied grid (~ 600 points per cm^2^) was a compromise between overall accuracy and workload.

#### Specific gravity

Ovary volume was estimated according to methodology described by Scherle^54^, which consisted of submerging the whole, intact ovary (Supplementary, Table S2) in a saline solution, presently with a specific gravity (ρ) of 1.007 g cm^−3^. The ovarian specific gravity for mackerel was thereafter determined from the ovary weight and volume of (early-) maturing, spawning, spent, and resting individuals, i.e. in stages 3 and 6–8 (see above; (Supplementary, Fig. S19A). No overall statistical difference existed (t-test, *p* = 0.746), although less so between the three last stages (t-test, *p* = 0.879) (Supplementary, Fig. S19B). Hence, the specific gravity for maturing stage (stages 3–5) was set at 1.020 g cm^−3^, and for spawning, spent, and recovering stages at 1.047 g cm^−3^.

#### Volume-based oocyte diameter

Oocyte diameter (OD_ij_) was averaged by manual measurements of short (S_ij_) and long (L_ij_) axes of three “through-the-nucleus-sectioned” oocytes per phase_i_ in each female (_j_), from PVO2 to GVBD, the last with disintegrating nucleus. This low number measured per oocyte phase is explained by the low variance in diameter within a given phase, though provided introducing a rich number of phases, each precisely defined^25^, as currently done (see above). Hydrated oocytes were not measured due to their irregular shape post histological dehydration. The shape factor (k_ij_) was individually estimated from the long and short axes ratio: k_ij_ = L_ij_/S_ij_. Then, phase_i_-specific mean volume-based oocyte diameter was given as:

$$OD_{Vij} = \left[ {\sum_{j = 1}^{ni} \left( {\frac{{(OD_{ij} )^{3} }}{{n_{i} }}} \right)} \right]^{\frac{1}{3}}$$

A correction factor was applied to rectify for oocyte shrinkage during histological processing (cODν_ij_)^22^.

### Fecundity measures

The number of oocytes produced in phase(_i_) in each ovary(_j_) (NO_ij_) was calculated from OPD_ij_ multiplied with formalin-preserved ovary weight (OW_j_), i.e. NO_ij_ = OPD_ij_ × OW_j_. The traditional body weight-based relative fecundity (RF_ij_) for each oocyte phase(_i_) and individual(_j_) was given as: RF_ij_ = NO_ij_/W_j_. The corresponding total length-based^28^ relative fecundity for each oocyte phase(_i_) and individual(_j_) was: RF_TLij_ = 100 × NO_ij_/TL^3.13^. The 3.13 refers to the slope from the length–weight relationship (see above and Supplementary, Fig. S7).

### Data analysis

All plots and statistical analyses were performed in R v4.0.4.^55^. Figures were produced by the packages: maps^56^, ggplot2^57^, with a few ggplot2 extensions such as ggridges^58^ and egg^59^.

## Supplementary Information

Supplementary Information 1.
