# Supplementary material for: First thorough assessment of de novo oocyte recruitment in a teleost serial spawner, the Northeast Atlantic mackerel (Scomber scombrus) case
Source: Sci Rep. 2021 Nov 8;11:21795. doi: 10.1038/s41598-021-01234-1 (PMC8575906; doi:10.1038/s41598-021-01234-1)
Supplement: Supplementary file 1 — Supplementary Information 1. [file 41598_2021_1234_MOESM1_ESM.pdf]

Supplementary material

First thorough assessment of de novo oocyte recruitment in a teleost serial spawner, the Northeast Atlantic mackerel (*Scomber scombrus*) case

Thassya C. dos Santos Schmidt[1,2*], Anders Thorsen[1], Aril Slotte[1], Leif Nøttestad[1], and Olav S. Kjesbu[1,2]

[1]Institute of Marine Research, PO Box 1870 Nordnes, NO-5817 Bergen, Norway

[2]Contributed equally to this work

[*]Correspondence author: T.C.d.S.S. (thassya@hi.no)

This file includes:

- Supplementary tables: Table S1 – S2
- Supplementary figures: Fig S1 – S19

Table S1. Histological features of different oocyte phases. The size range in phase-specific oocyte diameter is presented before (values inside square brackets) and after correction for shrinkage, the grand mean is also given (value inside the parentheses). Description of 'others' structures such as atresia and postovulatory follicles are also included. Scale bar (in µm) is shown in each image. Only oocytes sectioned through the nucleus were measured.

Oocyte phase	Picture	Features	Size range (µm)
Previtellogenic 1 (PVO1)	(this very small oocytes were presently held outside the study)	Small oval cell, uniform and homogeneous cytoplasm, large central nucleus	32 – 47 (38) [19 – 32 (24)]
Previtellogenic 2 (PVO2)	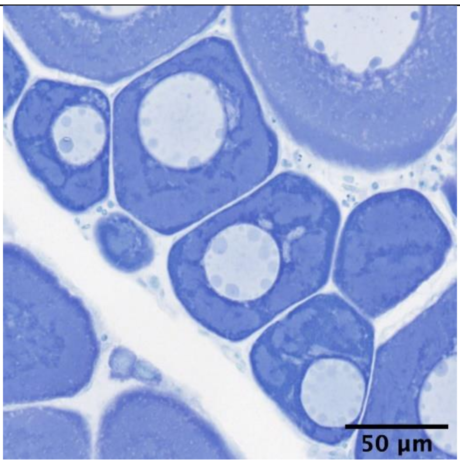	Cytoplasm stains densely and irregularly (light areas), large central nucleus, presence of multiple nucleoli	86 – 142 (112) [68 – 118 (91)]
Previtellogenic 3 (PVO3)	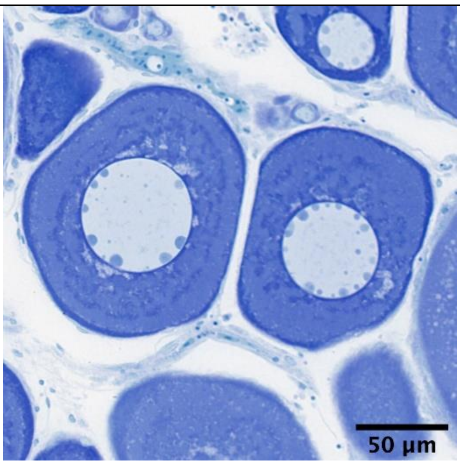	Cytoplasm stains more uniformly; there are small areas that stain more strongly	124 – 198 (158) [102 – 169 (132)]
Previtellogenic 4a (PVO4a)	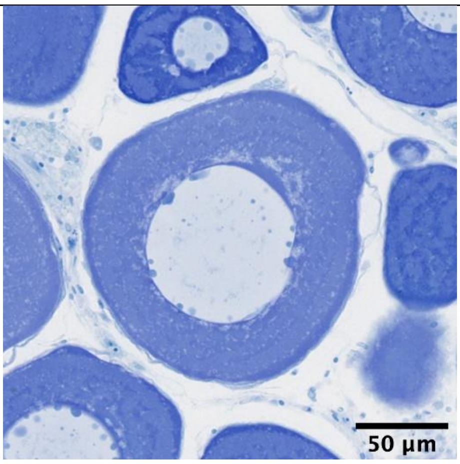	Presence of circumnuclear ring (CNR) that occupies almost the whole cytoplasm	162 – 228 (197) [136 – 196 (168)]

Previtellogenic 4b (PVO4b)	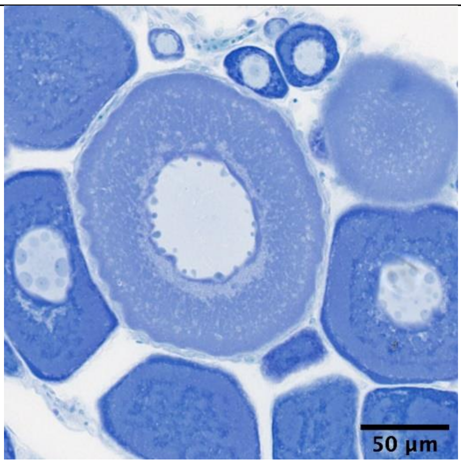	Distinct CNR separated from the nucleus by a homogeneous cytoplasm layer	184 – 266 (225) [157 – 231 (193)]
Previtellogenic 4c (PVO4c)	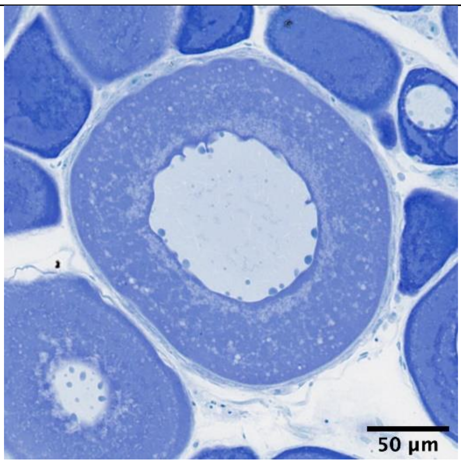	CNR gradually disappears and cytoplasm becomes more homogeneous	208 – 271 (243) [178 – 235 (210)]
Cortical alveoli (CA)	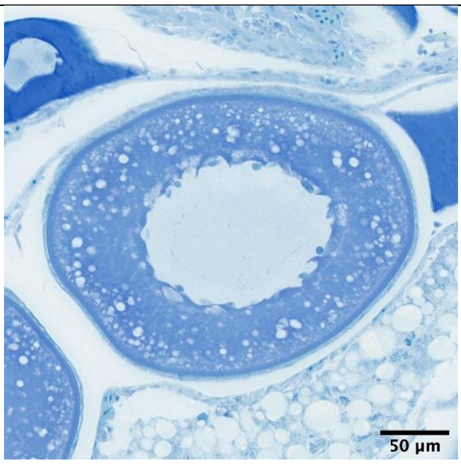	Cytoplasm vacuolated; chorion stains strongly	226 – 343 (291) [194 – 301 (253)]
Primary vitellogenic (VO1)	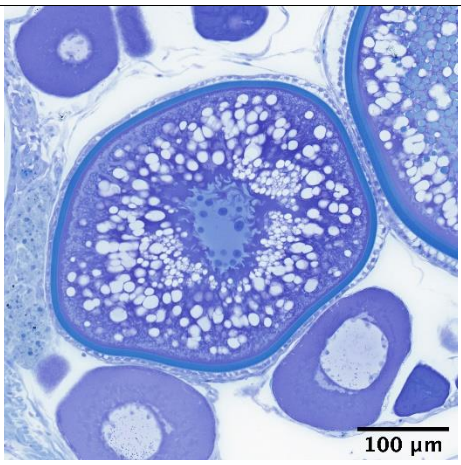	Presence of small yolk granules in the oocyte periphery or around the nucleus	379 – 418 (403) [276 – 445 (355)]

Secondary vitellogenic (VO2)	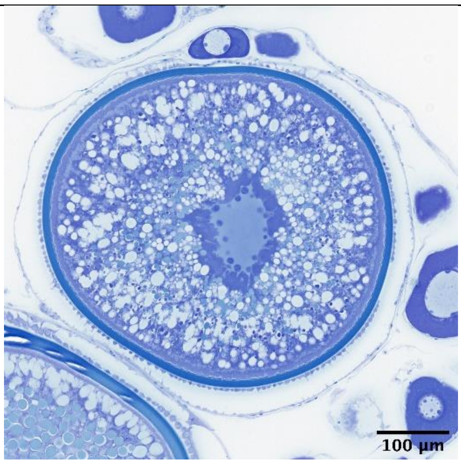	Larger yolk granules throughout the cytoplasm, inner chorion layer is thinner than outer chorion layer	416 – 595 (505) [366 – 528 (447)]
Tertiary vitellogenic (VO3)	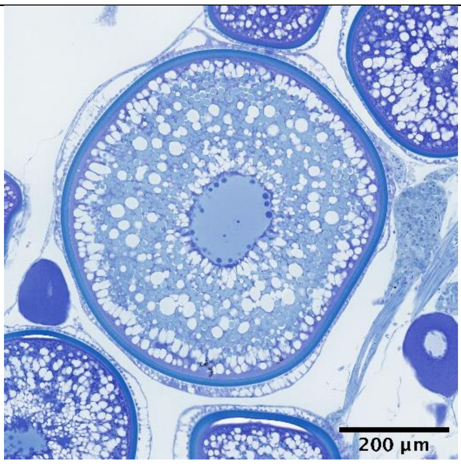	Yolk accumulation almost completed, oil droplets if present begin to surround the nucleus. Inner and outer chorion layer show same thickness	544 – 752 (663) [482 – 670 (590)]
Germinal vesicle migration (GVM)	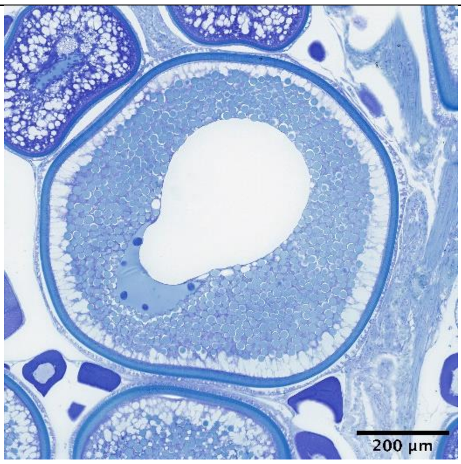	Oil droplets coalesce into a large single oil globule, nucleus migrates to the animal pole	676 – 863 (783) [601 – 771 (699)]
Germinal vesicle breakdown (GVBD)	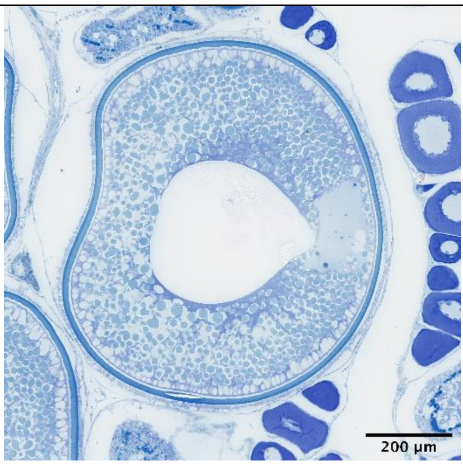	Germinal vesicle is not visible; homogeneous cytoplasm – yolk coalesce	777 – 1194 (924) [693 – 1071 (826)]

Hydrating (HYD)	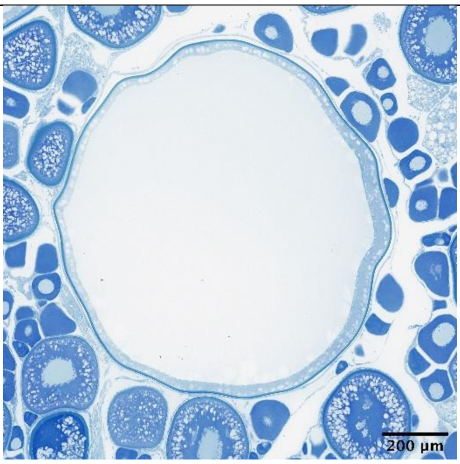	Large size due to water intake; can have irregular shape due to histological processing (dehydration), follicular layer still present, cytoplasm including cortical alveoli located in the periphery	
**‘Other’ structures**			
Postovulatory follicle (POF)	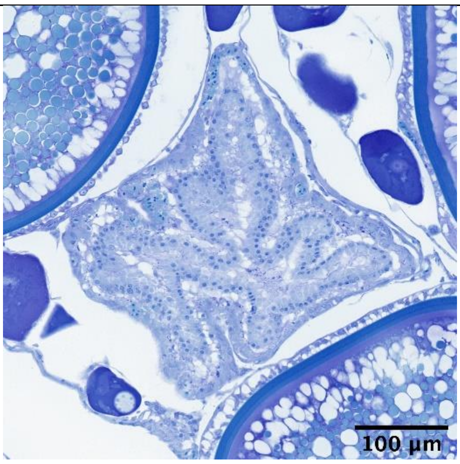	Collapsed follicle (i.e. irregular shape) from a spawned egg. Lumen is present in recent POF’s and absent in old POF’s	
Early alpha atresia (Eα atresia)	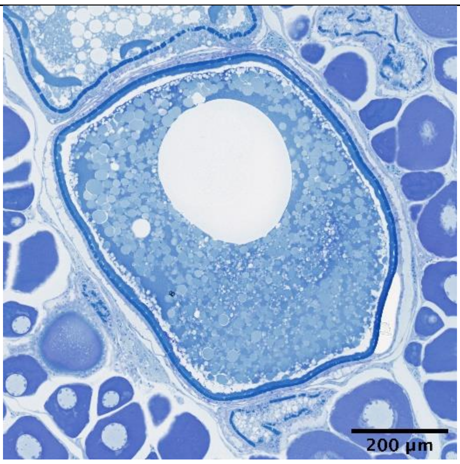	Hypertrophied follicle; broken chorion. Break in the chorion is smaller than the chorion thickness, disorganized cytoplasm	

Late alpha atresia (Lα atresia)	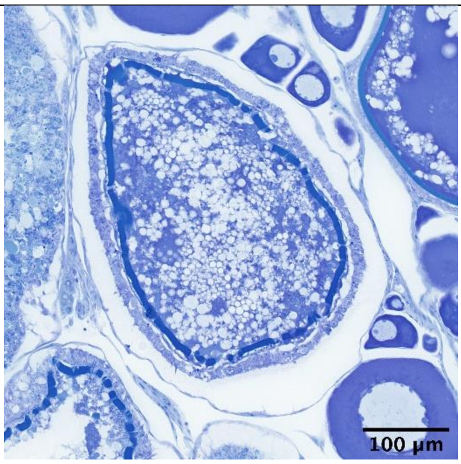	Hypertrophied/ smaller size follicle; broken, wrinkled, pitted or residual chorion, disorganized cytoplasm	
Atresia of previtellogenic oocytes (PVO)	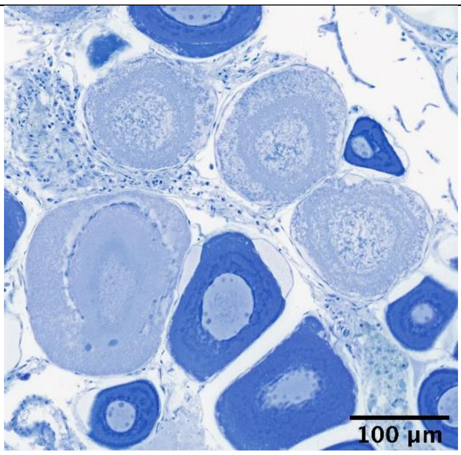	Compared to PVO's without atresia, cytoplasm in atretic PVO's stain weakly, nucleus disintegrates	
Beta atresia (β-atresia)	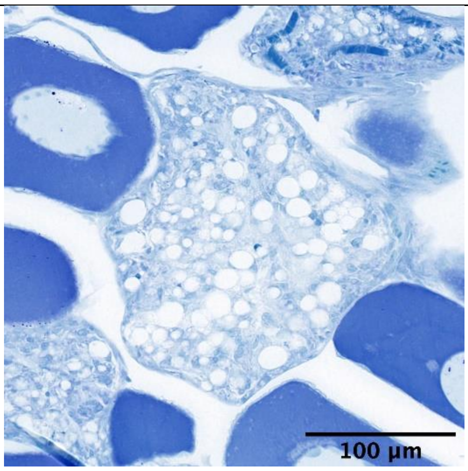	Chorion have disappeared. Small size, highly vacuolated and no visible yolk	

Table S2. Sampling and analyses overview**.** Summary of number of Northeast Atlantic mackerel females (N) caught and used for wholemount, histology, ovary specific gravity and oocyte package density (OPD) examinations by month in 2018 and 2019.

**Period**	**Season**	**Ovary samples**	**Wholemount analysis**	**Histology**	**Specific gravity**	**OPD**
4 - 15 May 2018	spawning	76	76	30	33	15
18 - 24 June 2018	spawning	84	84	30	25	12
6 - 30 July 2018	spawning	391	369	74	86	11
14 August 2018	feeding	21	21	15	8	10
15 September 2018	feeding	100	100	30	25	10
3 and 14 October 2018	wintering	200	200	30	57	11
13 and 25 November 2018	wintering	146	146	30	24	10
15 January 2019	wintering	96	96	29	21	10
29 - 31 March 2019	spawning	5	5	5	0	5
17 - 23 April 2019	spawning	51	51	30	0	15
5 - 25 May 2019	spawning	193	193	45	0	18
11 - 26 June 2019	spawning	220	220	56	0	17
**Total**		**1583**	**1561**	**404**	**279**	**144**

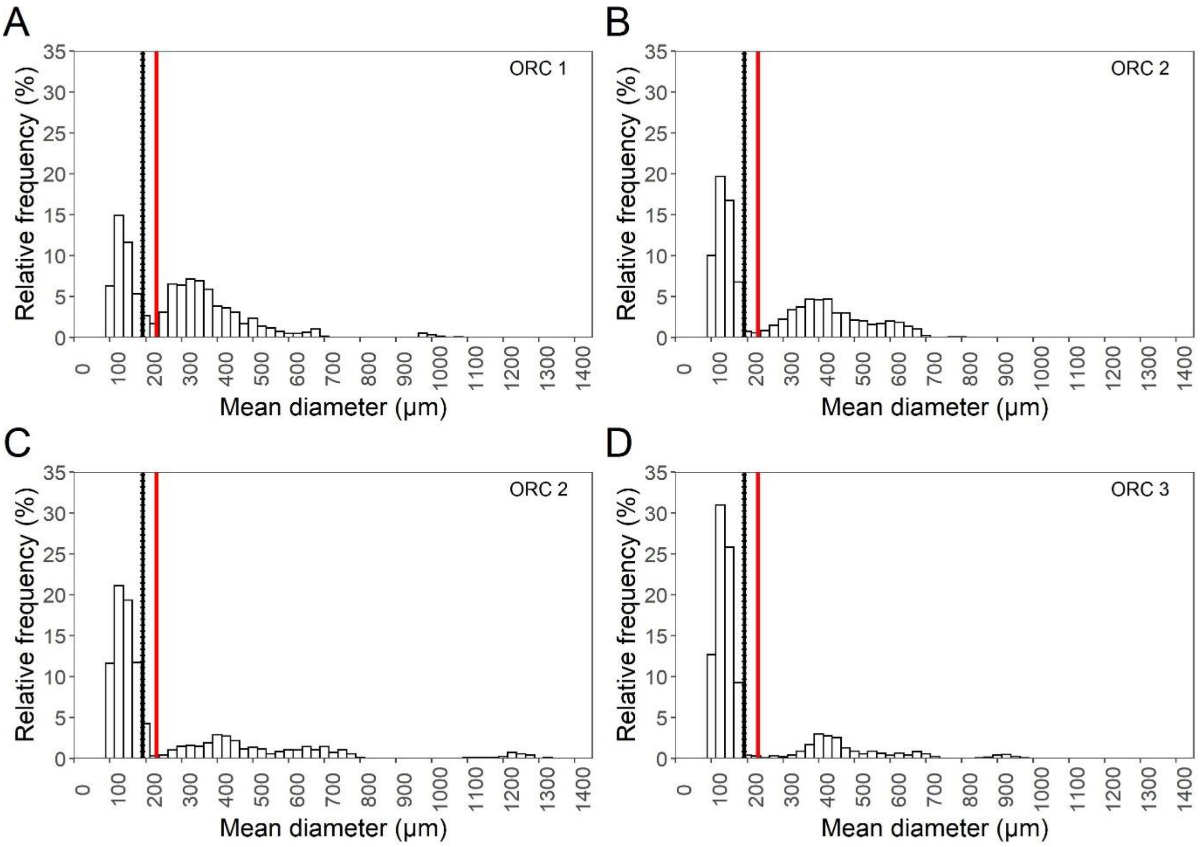

Fig. S1. Oocyte size frequency distribution and threshold between previtellogenic and cortical alveoli-vitellogenic oocytes. Oocyte size frequency distribution of four random mackerel females caught during the spawning season (A and B – female sampled in May 2019, C and D – June 2019). Black, thick line indicates the mean threshold (192 µm) and confidence bands (dashed points) found statistically based on the data distribution, whereas the red line shows the threshold estimated through histological analysis (oocyte phase PVO4c). The oocyte ratio category (ORC) for each individual is indicated.

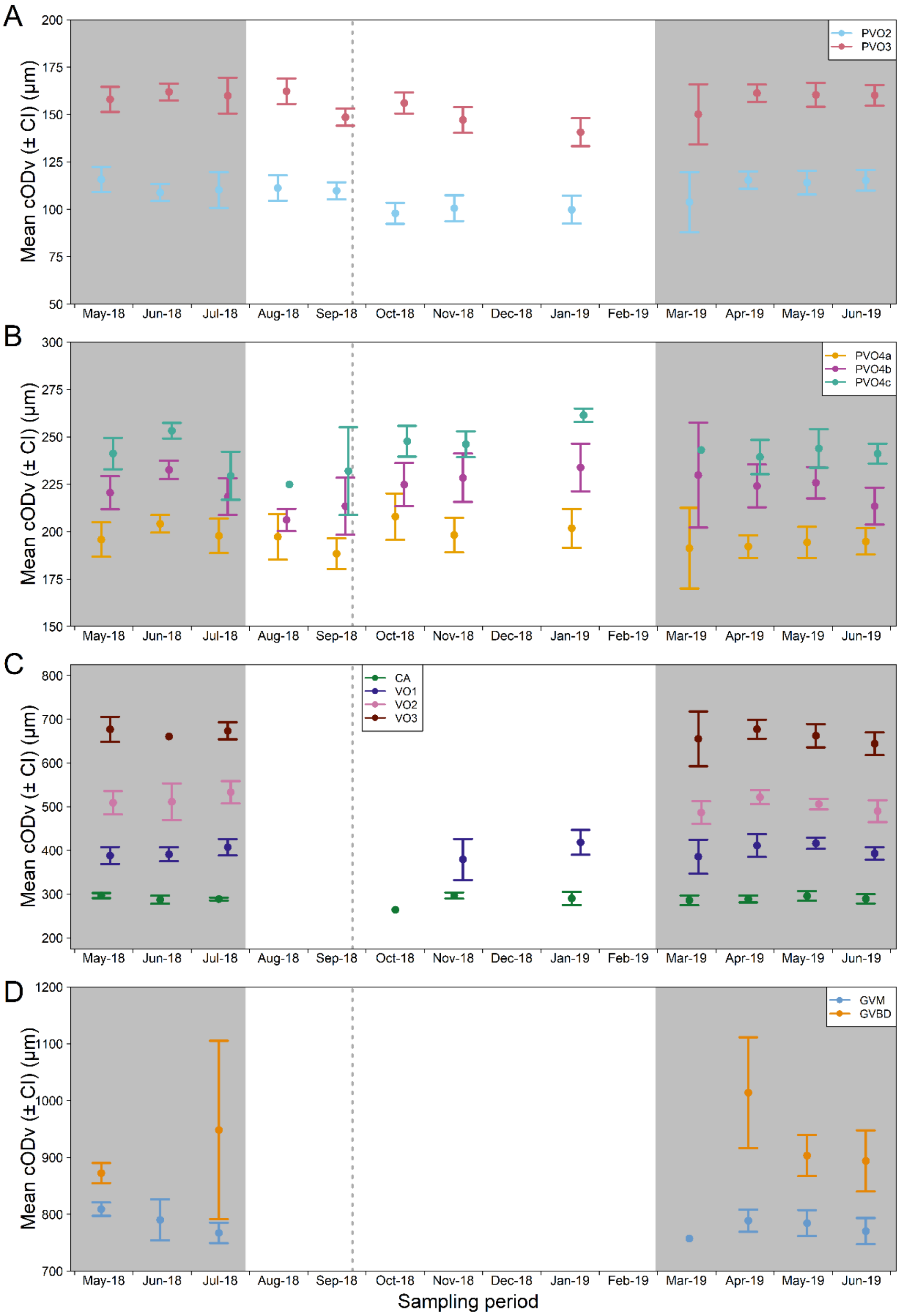

Fig. S2. Monthly variation in phase$_i$-specific volume-based mean oocyte diameter (cODv). (A) Monthly variation in cODv of small previtellogenic oocytes (PVO2 and PVO3); (B) large previtellogenic oocytes (PVO4a-c), (C) cortical alveoli oocytes (CA) and vitellogenic oocytes (VO1-3), and (D) oocytes in final maturation (germinal vesicle migration [GVM] and germinal vesicle breakdown [GVBD]). Vertical line indicates the autumn equinox. Note that the y-axis scale differs between panels. Grey bands indicate spawning period. No samples were collected in December-18 and February-19.

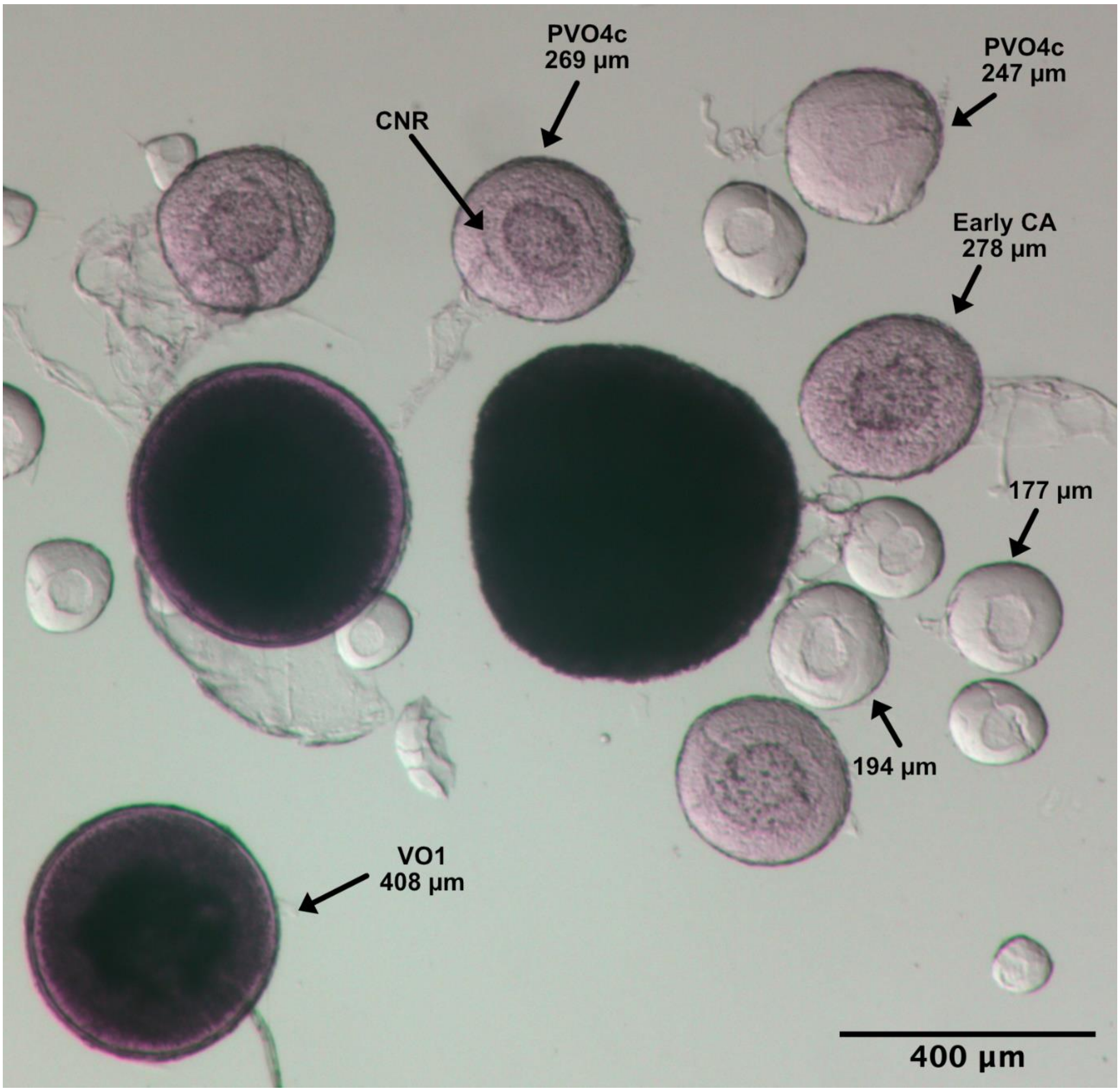

Fig. S3. Unstained wholemount preparations of oocytes. Examples of PVO4c, CA and VO1 with, in cases, annotated diameter. The ICES size threshold for fecundity counting is set at 185 μm^3. CNR: foreseen to be the circumnuclear ring. The size bar is included.

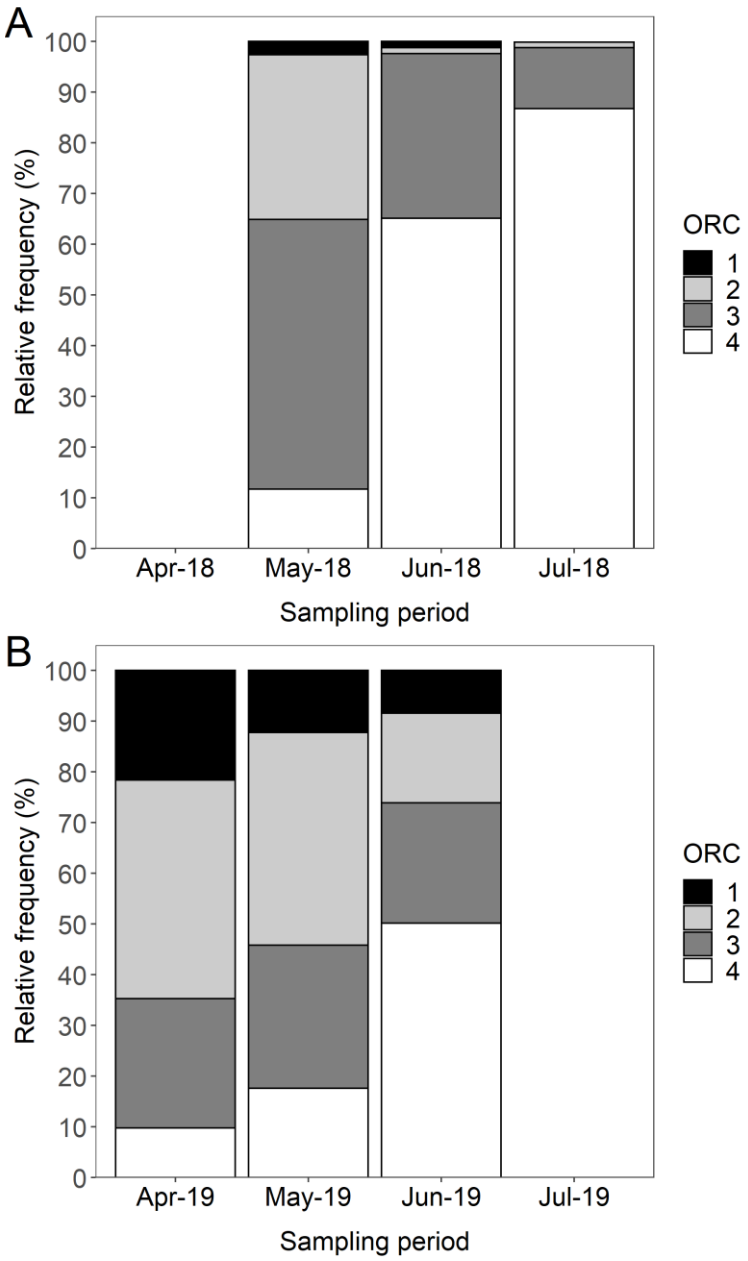

Fig. S4. Frequency of occurrence of oocyte ratio category (ORC) during the spawning season. ORC1 is early-spawning fish, ORC2 is mid-spawning fish, ORC3 is late-spawning fish, and ORC4 is very late- or post-spawning fish. No samples were collected in April-18 and July-19.

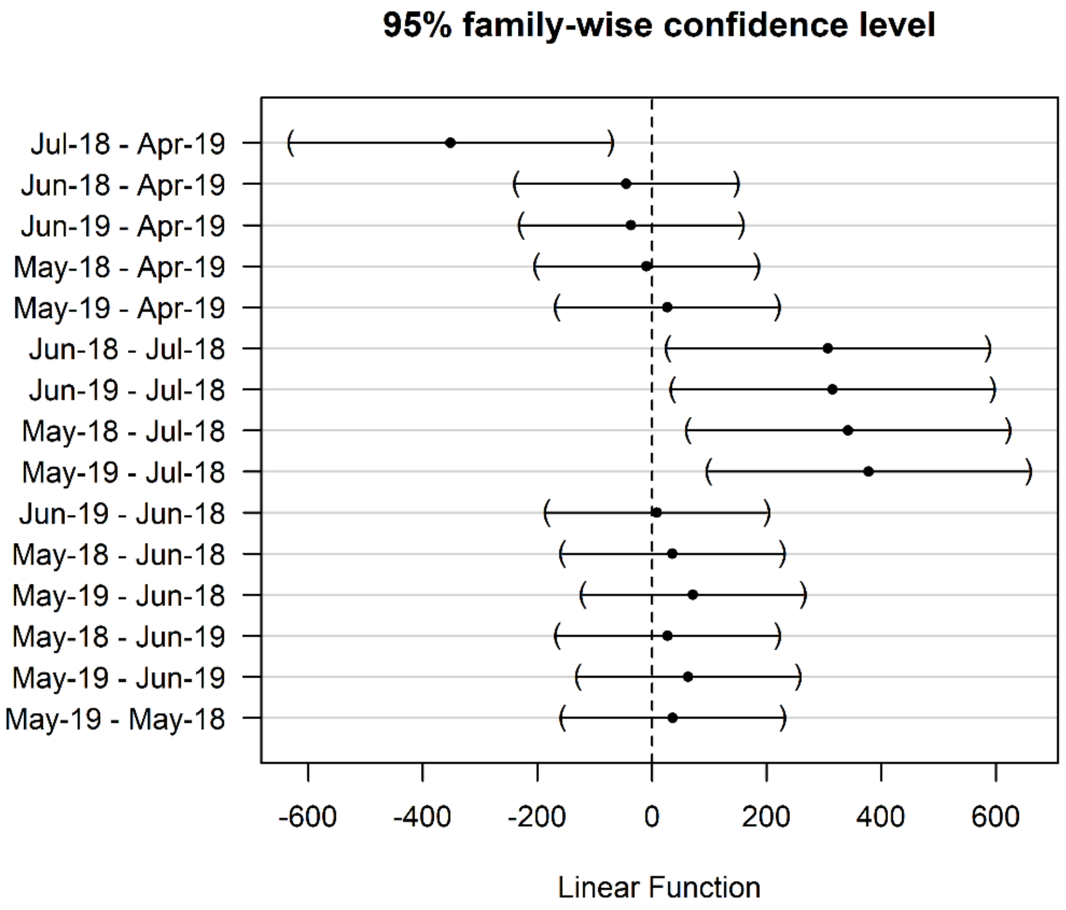

Fig. S5. *A posteriori* Tukey test. Comparison between oocyte ratio category (ORC) distribution among sampling periods. Mean and 95% confidence interval are shown, with dashed line indicating no difference.

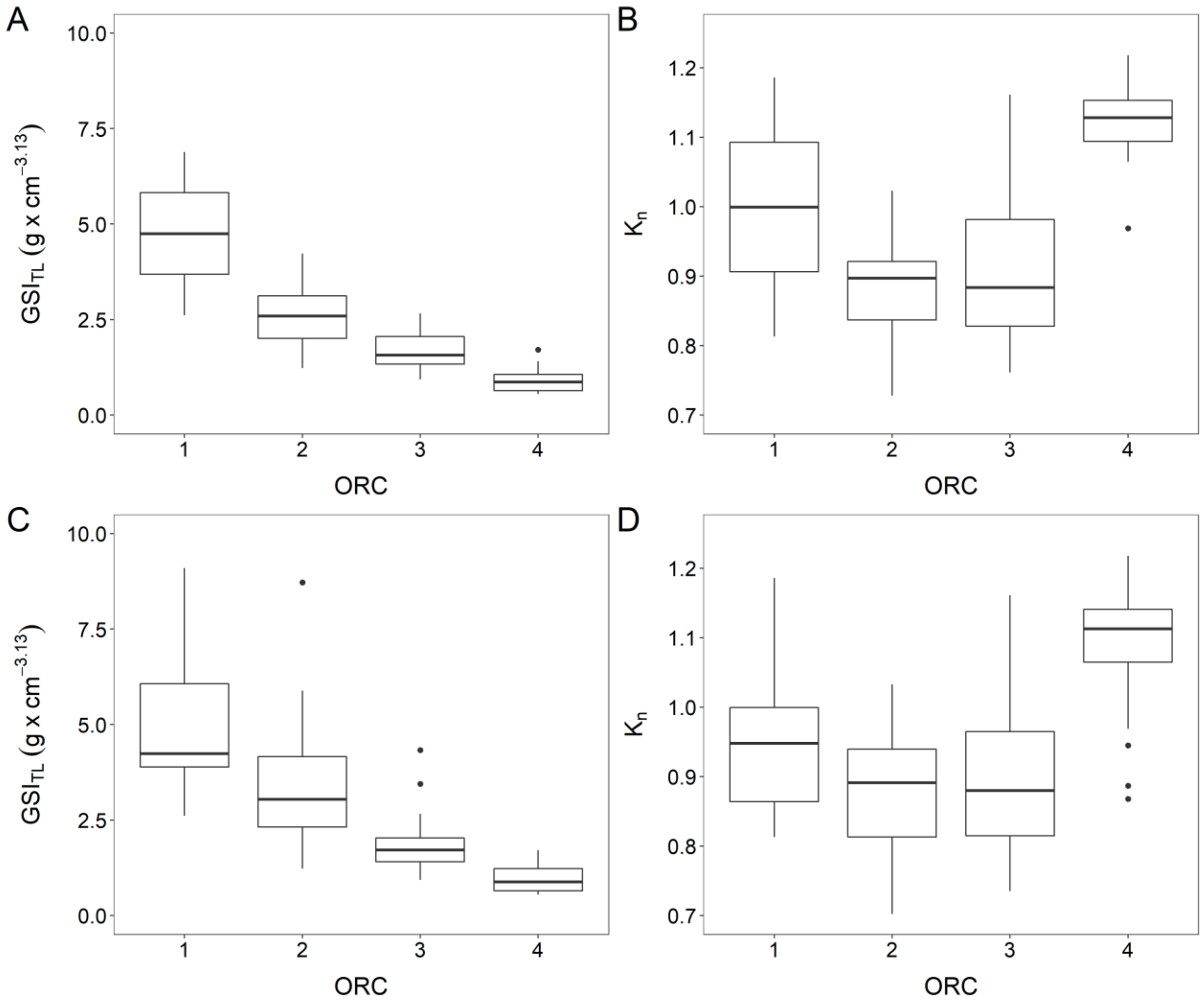

Fig. S6. Length-based gonadosomatic index (GSI_{TL}) (A and C) and relative condition (K_n) (B and D) as a function of stage of spawning, represented by ORC. Top panels A and B represent oocyte packing density (OPD) samples collected in 2018, and bottom panels C and D those sampled in 2019 (Supplementary, Table S2). For each box plot, the thick line is the median value, top and bottom lines indicate the 75th and 25th percentiles, respectively, and whiskers indicate maximum and minimum value.

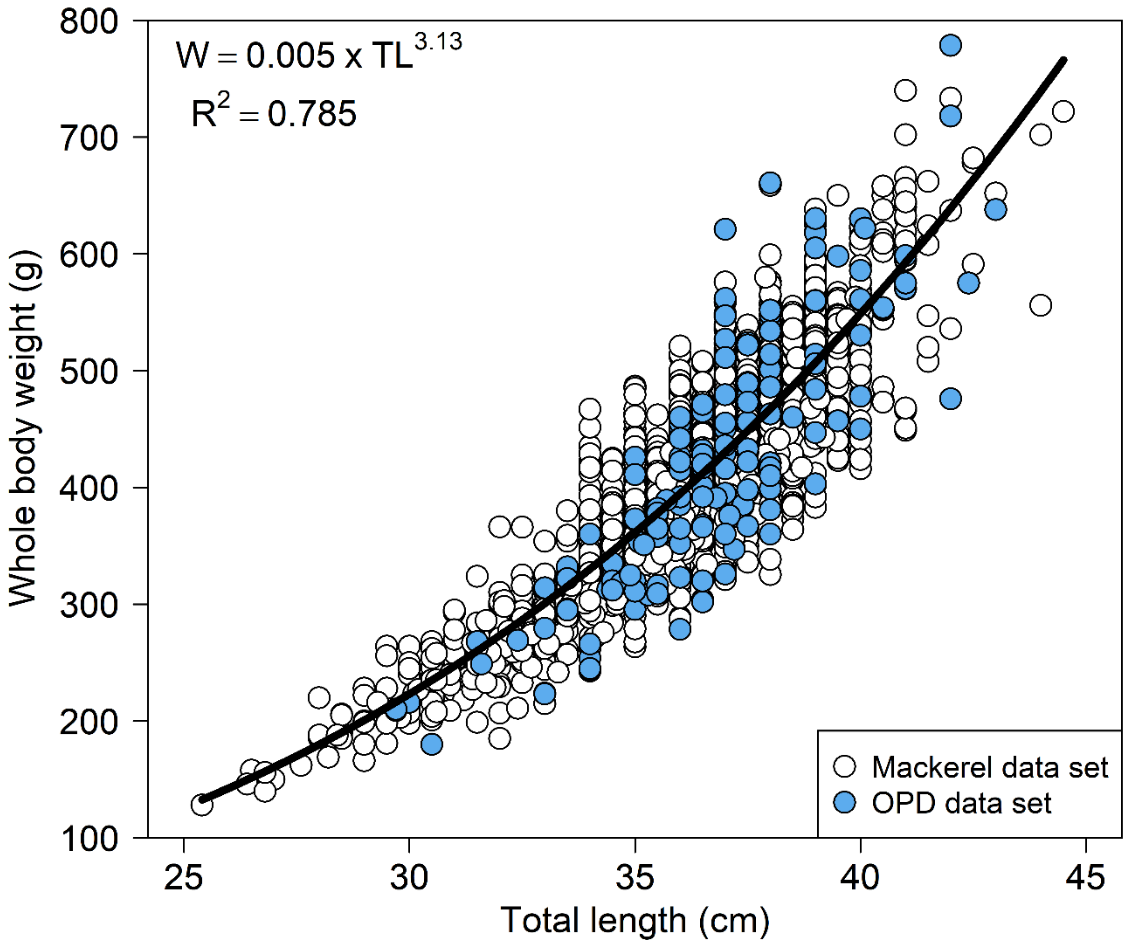

Fig. S7. Length-weight relationship. Relationship between weight and length of all mackerel females collected from May 2018 to June 2019. The oocyte packing density (OPD) theory data subset is specially identified. The curve was based on the power function: $W = a \times TL^b$.

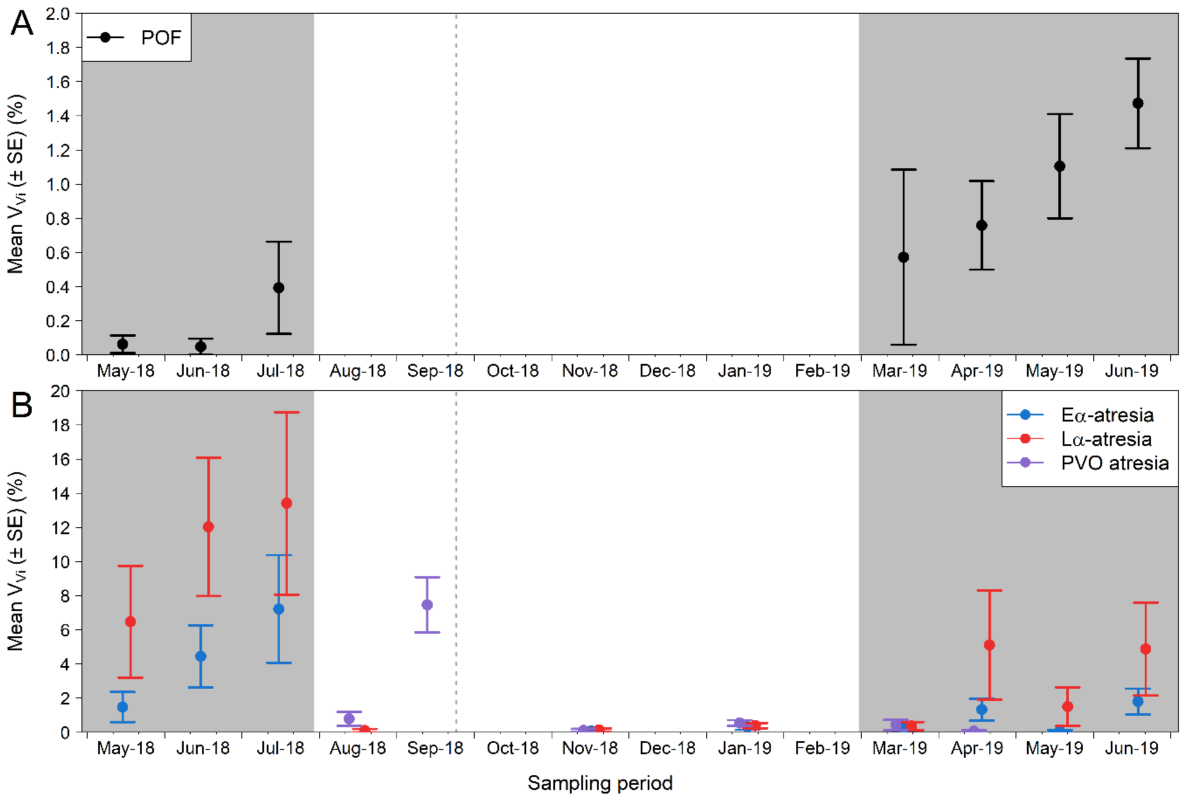

Fig. S8. Monthly variation in volume fraction of (A) postovulatory follicles (POF) and (B) different atresia stages. The autumn equinox is indicated (vertical dashed line). Grey bands indicate the spawning season. Note that the y-axis scale differs between panels. No samples were collected in December-18, and February-19.

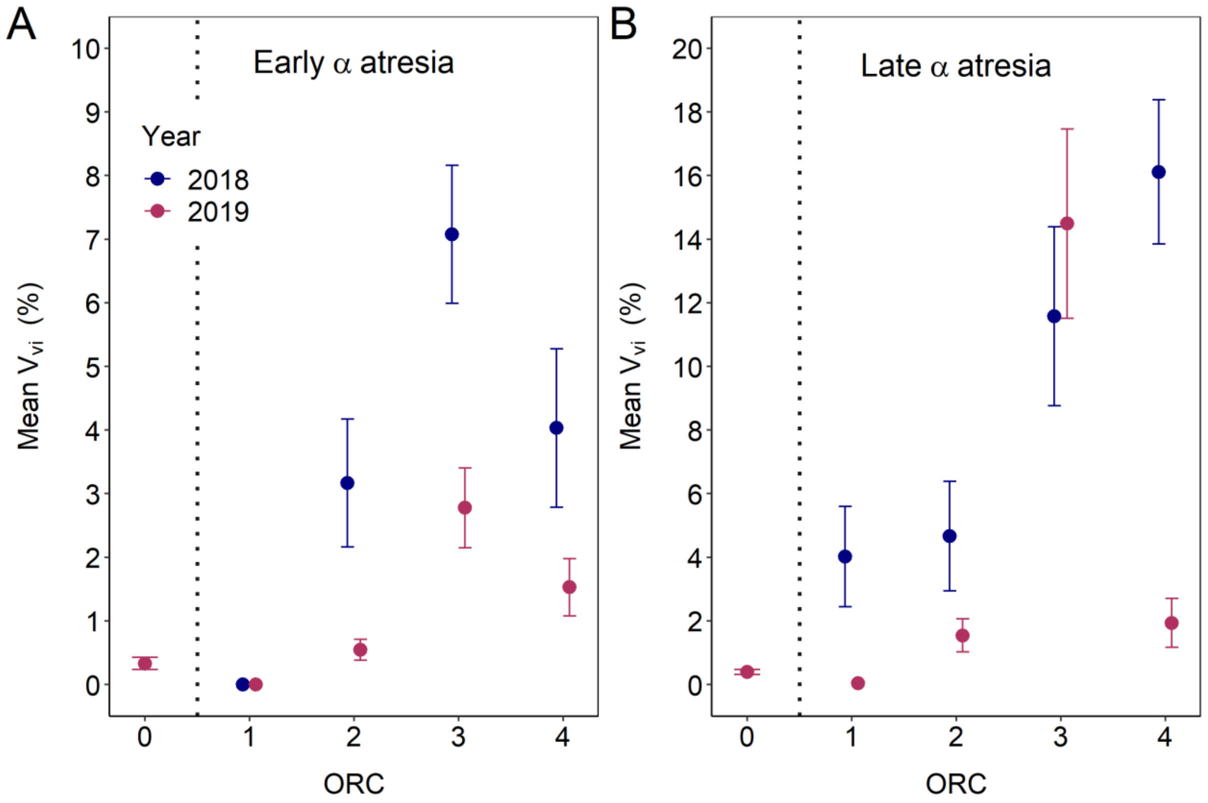

Fig. S9. Alpha atresia throughout spawning. Mean volume fraction of early (A) and late (B) alpha (α) atresia according to oocyte ratio category (ORC) in both years. Vertical line separates prespawning individuals (ORC0, January) from those that have already started spawning (ORC1 to 4). Note that the y-axis scale differs between panels.

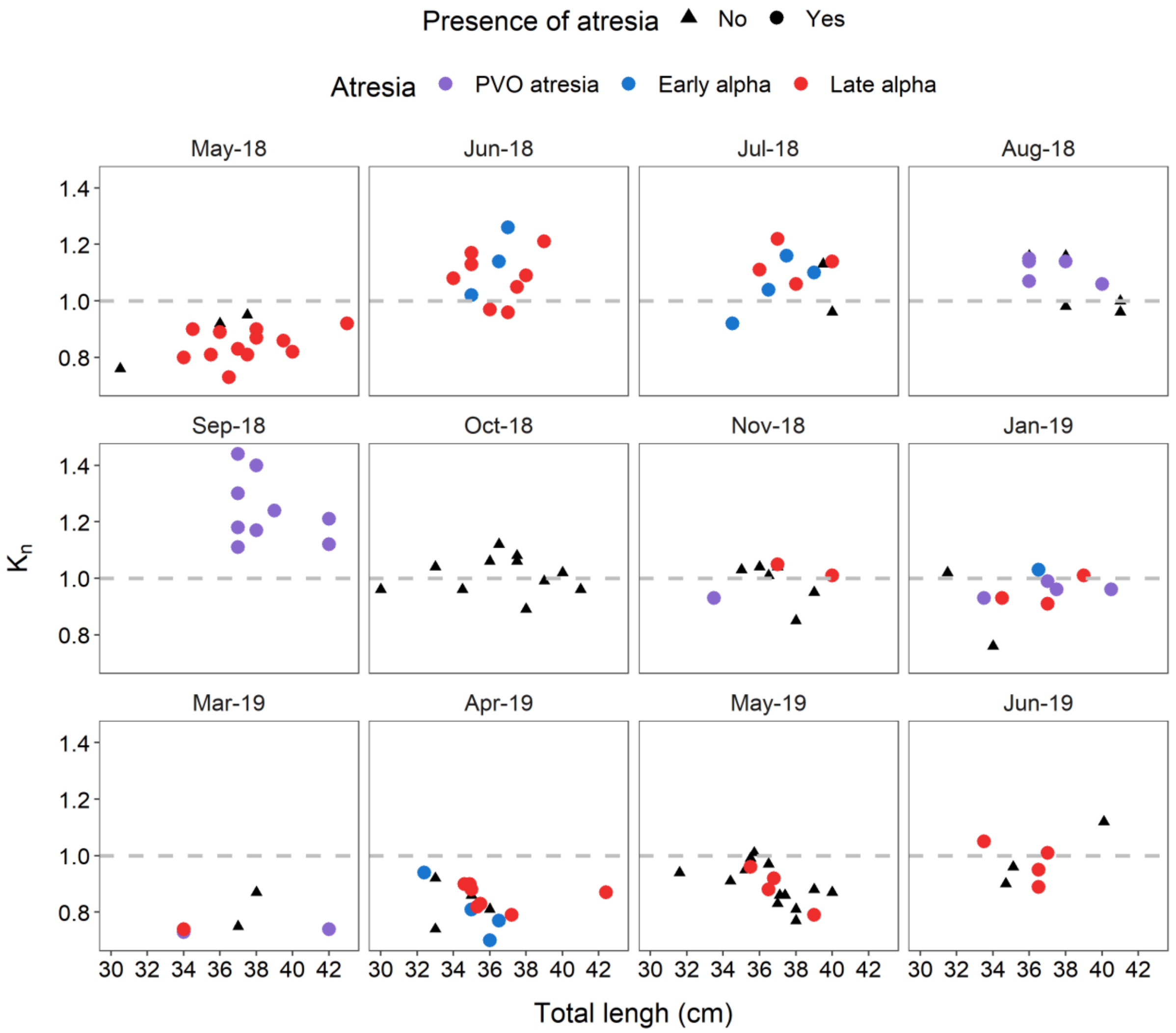

Fig. S10. Atresia versus fish condition by month. Relationship between relative condition (K_n) and total length grouped by atresia stages over the sampling period. Samples without atresia are also presented. Dashed horizontal line indicates the threshold between high and low fish body condition[1]. Only females used in the oocyte packing density (OPD) method are included (Supplementary, Table S2).

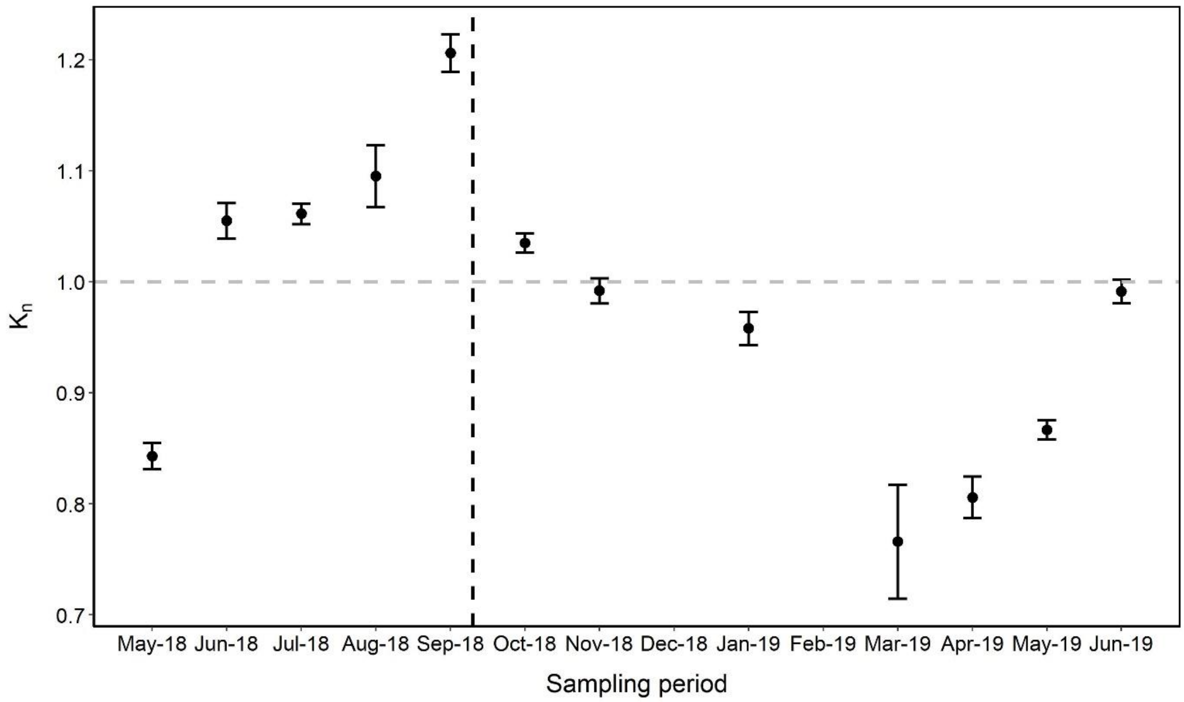

Fig. S11. Monthly relative condition. Mean (± 95% confidence interval) relative condition per month for all mackerel female samples collected from May 2018 to June 2019. Vertical line indicates the autumn equinox, and the horizontal line, the threshold between high and low fish body condition[1]. No samples were collected in December-18 and February-19.

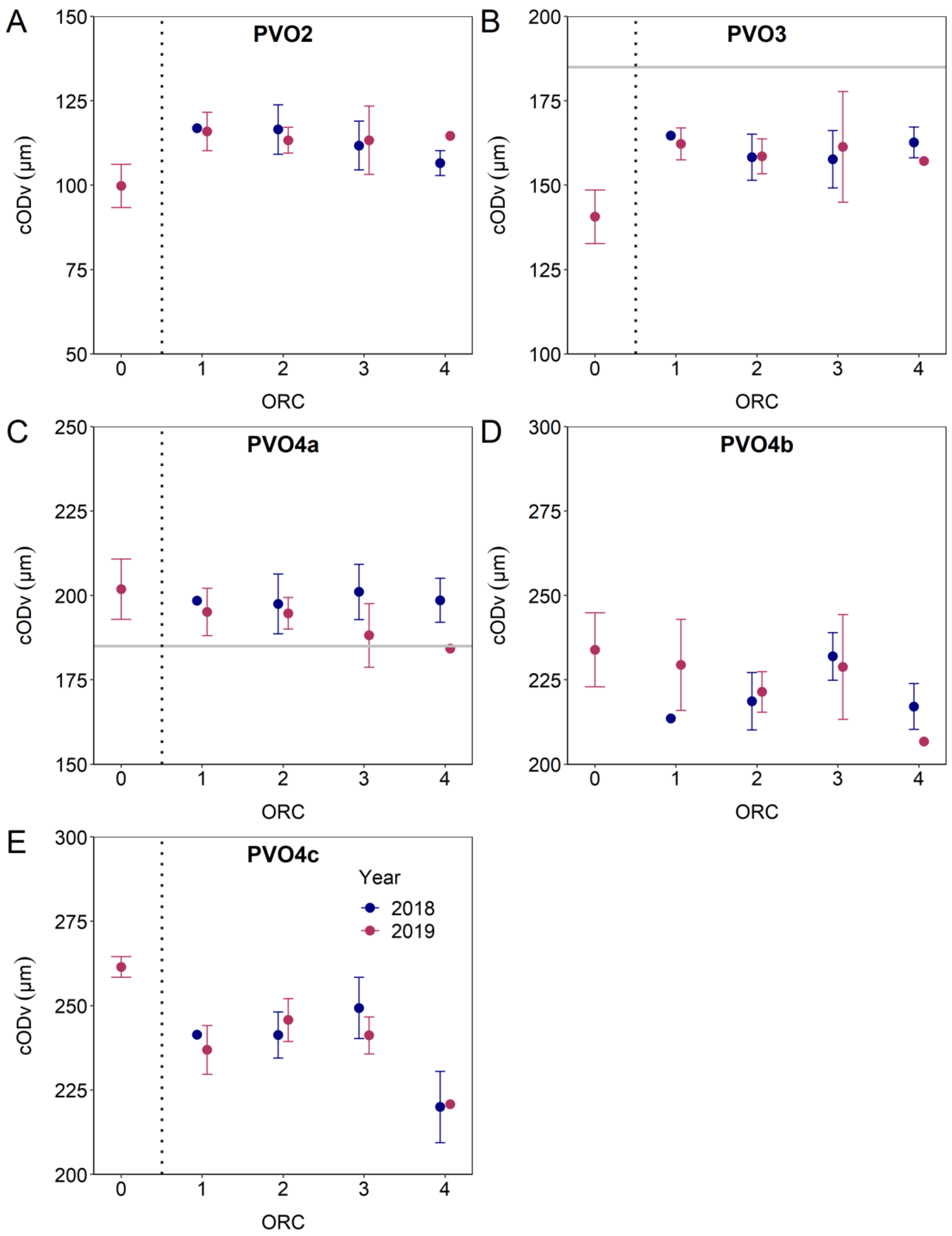

Fig. S12. Diameter of previtellogenic oocytes by oocyte ratio category. Mean (± 95% confidence interval) oocyte diameter (cODv) of phases PVO2 to PVO4a-c by oocyte ratio category (ORC) in 2018 and 2019. Vertical line separates prespawning individuals (ORC0, January) from those that have already started spawning (ORC1 to 4). Horizontal line refers to the ODv at 185 µm (as estimated by Greer-Walker et al.[2] and used in ICES mackerel fecundity estimates[3]). Note that the y-axis scale differs between panels.

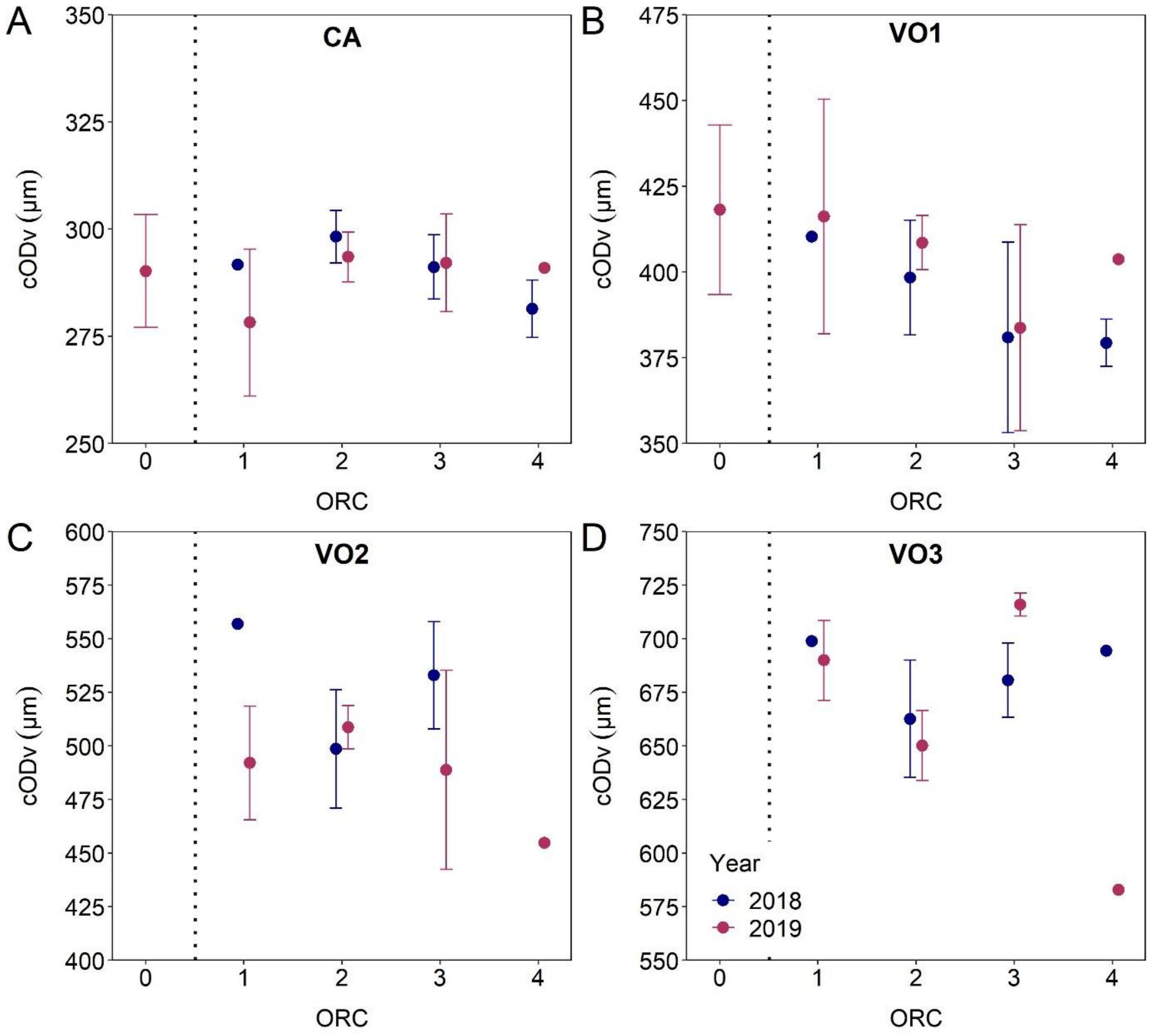

Fig. S13. Diameter of cortical alveoli and vitellogenic oocytes by oocyte ratio category. Mean (± 95% confidence interval) oocyte diameter (cODv) of CA and VO1-3 by oocyte ratio category (ORC) in 2018 and 2019. Vertical line separates prespawning fish (ORC0, January) from fish that has already started spawning (ORC1 to 4). Note that the y-axis scale differs between panels.

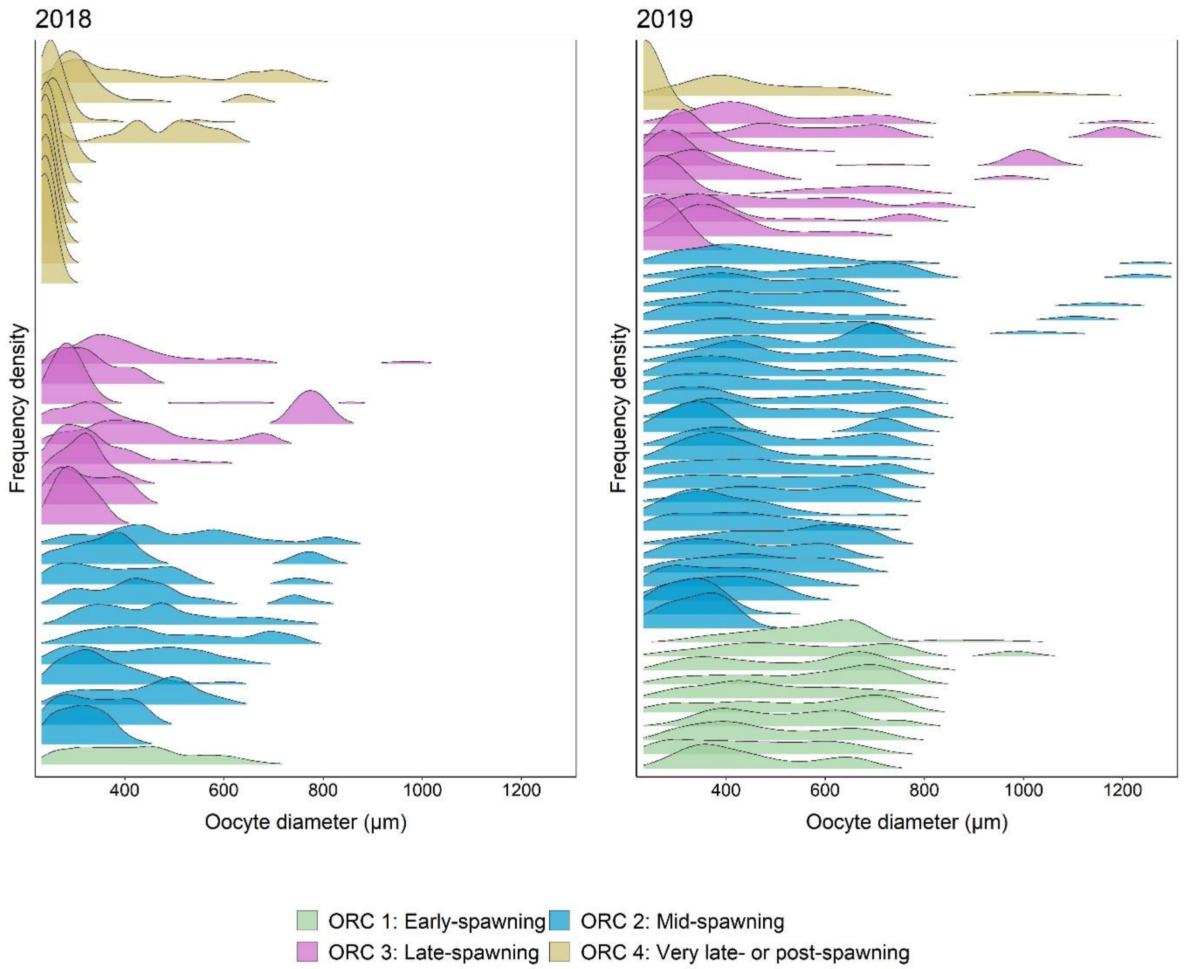

Fig. S14. Smoothed oocyte size frequency distribution (OSFD) for each female, with oocyte diameter ≥ 230 µm, according to oocyte ratio category. OSFD is organized in ascending order based on the maximum oocyte diameter within each ORC, all wholemount measurements. Note that a slight change in the smooth curve may occurred due the exclusion of oocytes smaller than 230 µm. The material refers to females subsequently examined for phase-specific oocyte packing density (OPD).

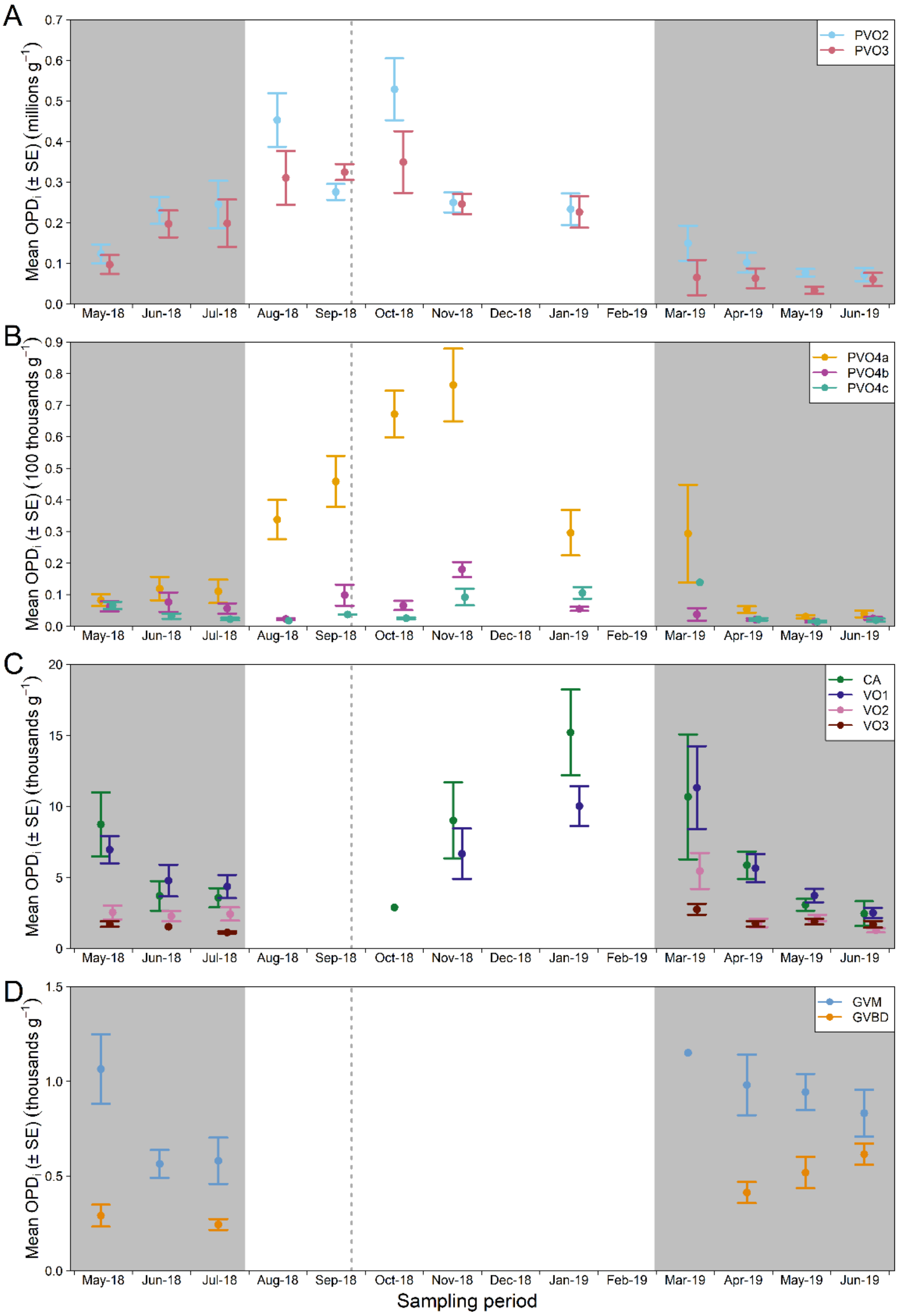

Fig. S15. Monthly variation in phase$_i$-specific oocyte packing density (OPD_i). (A) Monthly variation in OPD_i of small previtellogenic oocytes (PVO2 and PVO3); (B) large previtellogenic oocytes (PVO4a-c), (C) cortical alveoli oocytes (CA) and vitellogenic oocytes (VO1-3), and (D) final oocyte maturation (germinal vesicle migration [GVM] and germinal vesicle breakdown [GVBD]). The autumn equinox is indicated (vertical dashed line). Note that the y-axis scale differs between panels. No samples were collected in December-18 and February-19.

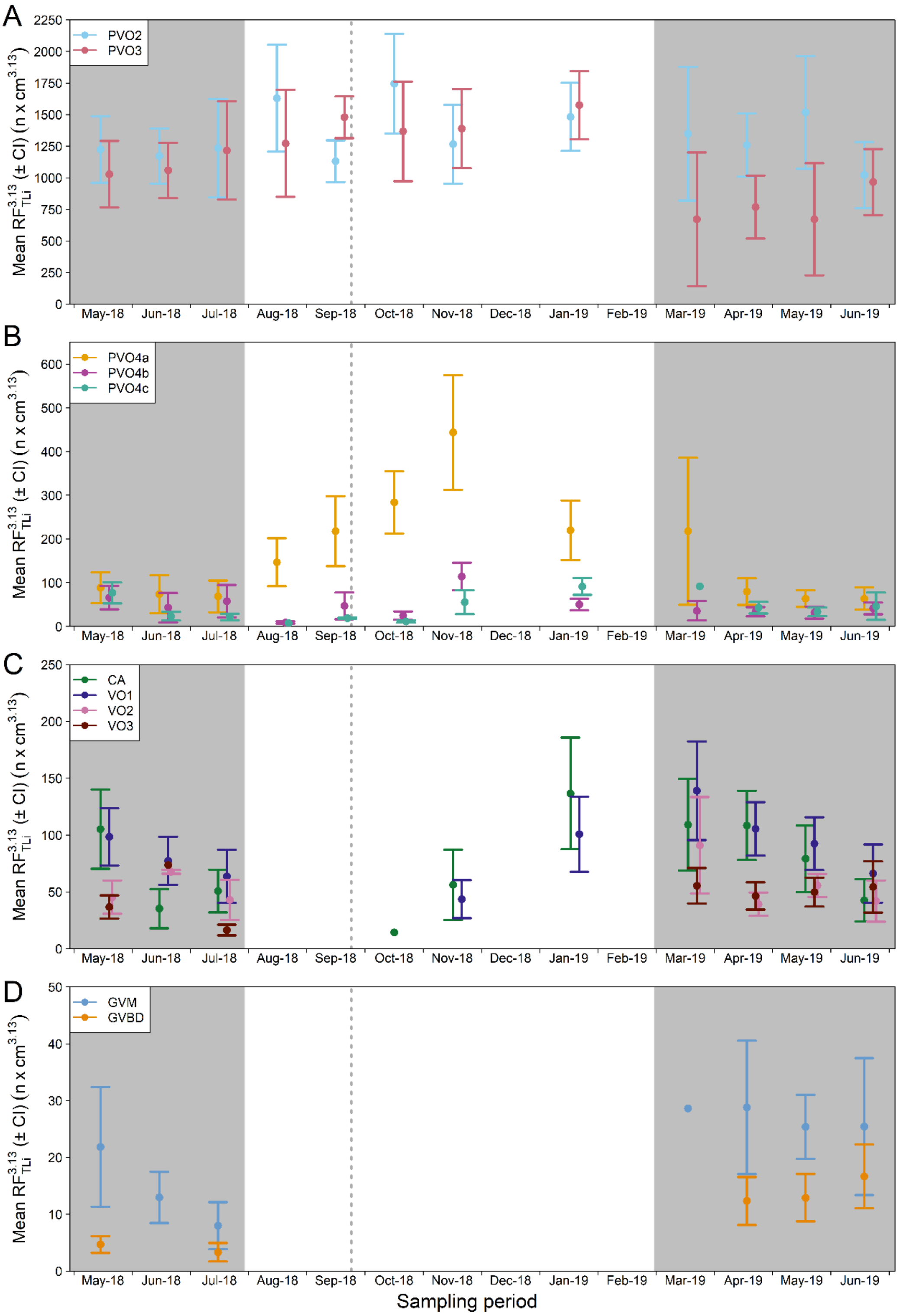

Fig. S16. Monthly variation in $phase_i$-specific relative length-based fecundity ($RF_{TLi}^{3.13}$). (A) Monthly variation in $RF_{TLi}^{3.13}$ of small previtellogenic oocytes (PVO2 and PVO3); (B) large previtellogenic oocytes (PVO4a-c), (C) cortical alveoli oocytes (CA) and vitellogenic oocytes (VO1-3), and (D) final oocyte maturation (germinal vesicle migration [GVM] and germinal vesicle breakdown [GVBD]). The autumn equinox is indicated (vertical dashed line). Note that the y-axis scale differs between panels. No samples were collected in December-18 and February-19.

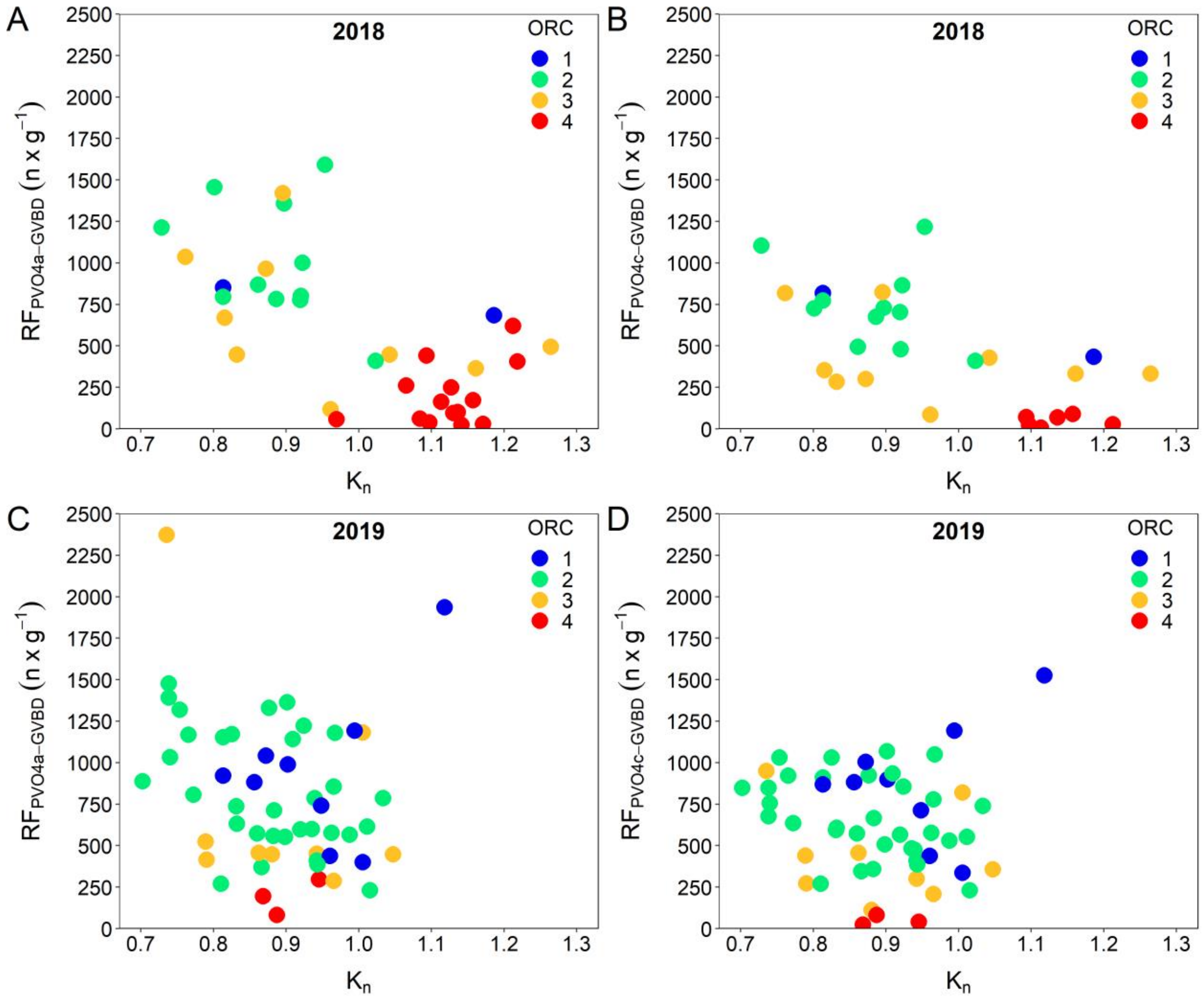

Fig. S17. Effect of relative condition (K_n) on relative fecundity. Individual relative fecundity was either estimated from PVO4a to GVBD ($RF_{PVO4a\text{-}GBVD}$), i.e. using 185 µm oocyte diameter as threshold[3] (A and C), or from PVO4c to GVBD ($RF_{PVO4c\text{-}GBVD}$), i.e. sum of oocytes from 230 µm (B and D) in each oocyte ratio category (ORC) in 2018 and 2019.

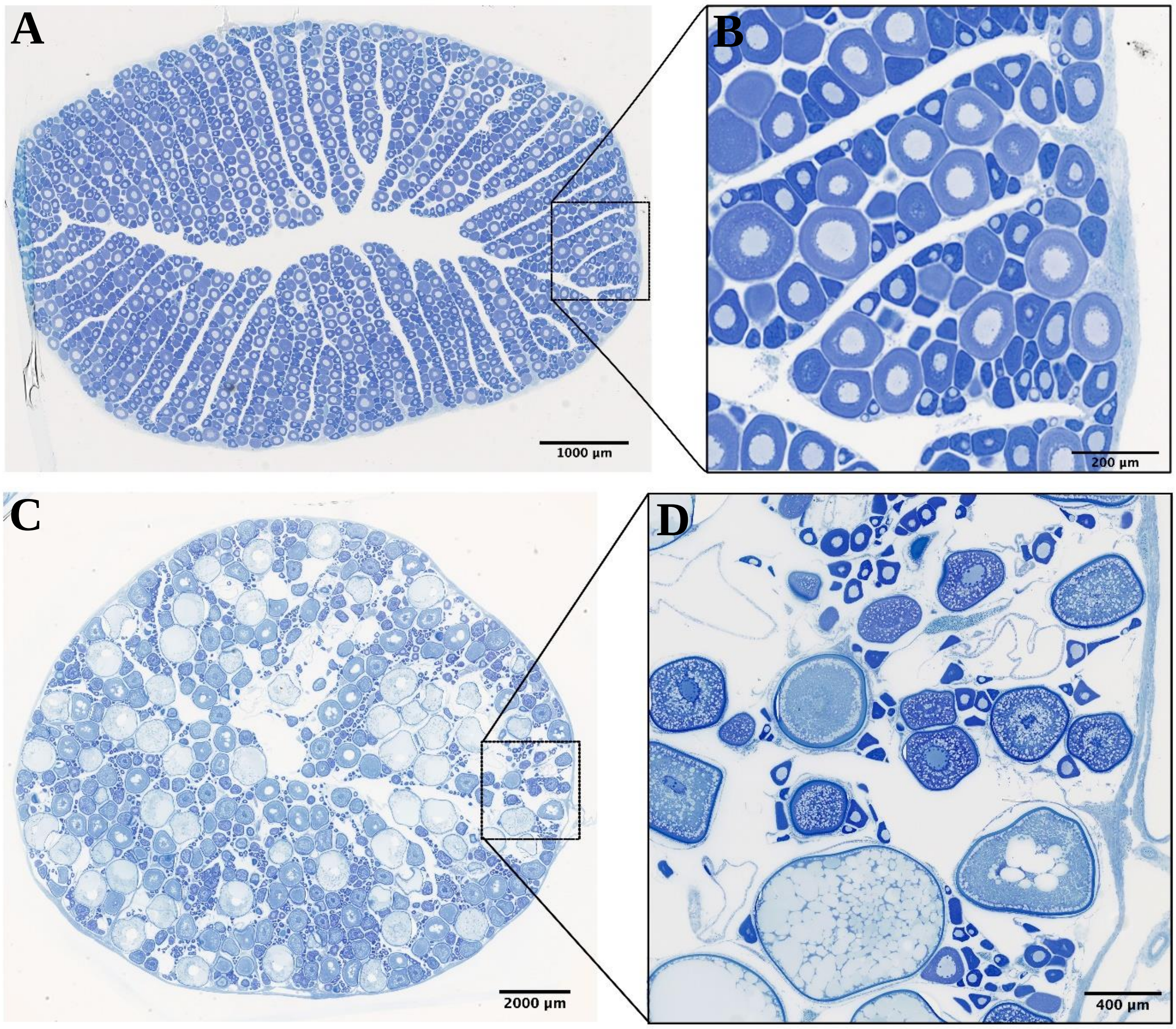

Fig. S18. Photomicrographs of a transverse section of the ovary in different stages of development: (A and B) previtellogenesis, and (C and D) final oocyte maturation. The insert (B and D) is a zoom-in view of a selected sub-area (box in A and C).

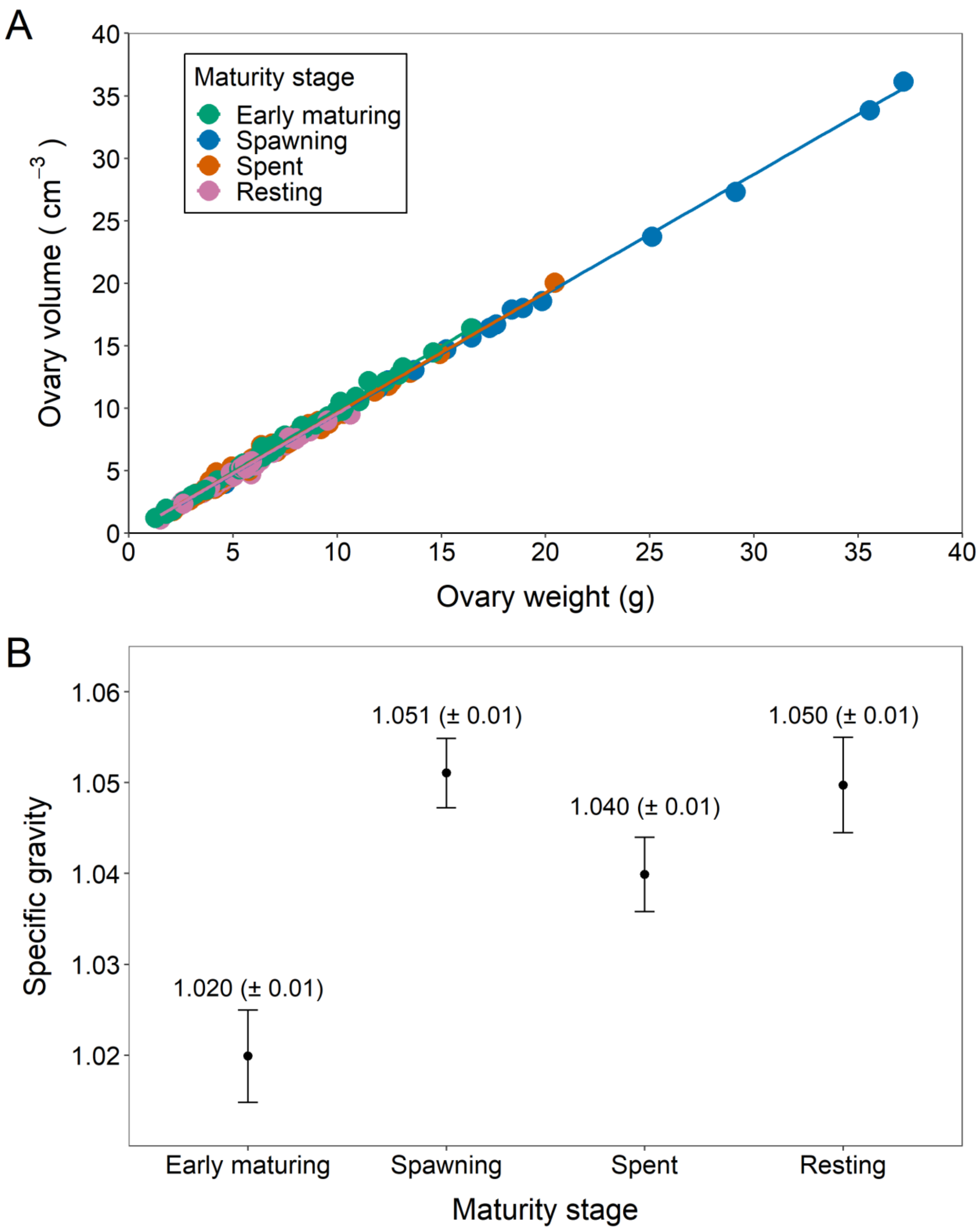

Fig. S19. Ovary volume and specific gravity. (A) Relationship between ovary volume and ovary weight split by maturity stage. (B) Mean and confidence interval (±CI) of ovarian specific gravity among maturity stages.

References

1 Le Cren, E. D. The length weight relationship and seasonal cycle in gonad weight and condition in the perch (*Perca flutuatilis*). *J. Anim. Ecol.*, 20, 201-219 (1951).

2 Greer-Walker, M., Witthames, P. R. & Bautista de los Santos, I. Is the fecundity of the Atlantic mackerel (*Scomber scombrus:* Scombridae) determinate? *Sarsia* **79**, 13-26 (1994).

3 ICES. ICES Working Group on Mackerel and Horse Mackerel Egg Surveys (WGMEGS: outputs from 2020 meeting). 88 (2021).
